# Parallel transmit 7T MRI for adult epilepsy pre‐surgical evaluation

**DOI:** 10.1111/epi.18353

**Published:** 2025-03-20

**Authors:** Krzysztof Klodowski, Minghao Zhang, Jian P. Jen, Daniel J. Scoffings, Robert Morris, Victoria Lupson, Franck Mauconduit, Aurélien Massire, Vincent Gras, Nicolas Boulant, Christopher T. Rodgers, Thomas E. Cope

**Affiliations:** ^1^ Wolfson Brain Imaging Centre University of Cambridge Cambridge UK; ^2^ Cambridge University Hospitals NHS Foundation Trust Cambridge UK; ^3^ Commissariat à l'Energie Atomique, CNRS, NeuroSpin, BAOBAB Université Paris‐Saclay Gif sur Yvette France; ^4^ Siemens Healthcare SAS Courbevoie France; ^5^ Department of Clinical Neurosciences University of Cambridge Cambridge UK; ^6^ MRC Cognition and Brain Sciences Unit University of Cambridge Cambridge UK

**Keywords:** 7T MRI, epilepsy surgery, focal epilepsy, parallel transmit

## Abstract

**Objective:**

To implement parallel transmit (pTx) 7T magnetic resonance imaging (MRI) in the pre‐surgical evaluation of 3T‐negative patients with drug‐resistant focal epilepsy, and to compare quality to conventional single transmit (specifically, circularly polarized [CP]) 7T MRI.

**Methods:**

We implemented a comparative protocol comprising both pTx and CP 7T MRI in consecutive adult candidates for epilepsy surgery who had negative or equivocal 3T MRI imaging. Here we report the outcomes from the first 31 patients. We acquired pTx and CP T_1_, T_2_, fluid‐attenuated inversion recovery (FLAIR) and edge‐enhancing gradient echo (EDGE) images, all in the same three‐dimensional (3D) 0.8 mm isotropic space. Two‐dimensional (2D) high‐resolution T_2_ and T_2_*‐weighted sequences were acquired only in CP mode due to current technological limitations. Two neuroradiologists, a neurologist, and a neurosurgeon made independent, blinded quality and preference ratings of pTx vs CP images. Quantitative methods were used to assess signal dropout.

**Results:**

7T revealed previously‐unseen structural lesions in nine patients (29%), confirmed 3T‐equivocal lesions in four patients (13%), and disproved 3T‐equivocal lesions in four patients (13%). Lesions were better visualized on pTx than CP in 57% of cases, and never better visualized on CP. Clinical management was altered by 7T in 18 cases (58%). Nine cases were offered surgical resection and one laser interstitial thermal therapy (LITT). Three cases were removed from the surgical pathway because of bilateral or extensive lesions. Five cases were offered stereo‐electroencephalography (sEEG) with better targeting (in three because the 7T lesion was deemed equivocal by the multi‐disciplinary team (MDT), and in two because the lesion was extensive). Blinded comparison confirmed significantly better overall quality of pTx FLAIR images (*F*(2, 184) = 13.7, *p* = 2.88 × 10^−6^), whereas pTx MP2RAGE images were subjectively non‐inferior and had improved temporal lobe coverage with quantitatively less signal drop‐out.

**Significance:**

pTx‐7T is implementable in a clinical pathway, changed management in 58% of patients where 3T + FDG‐PET had not enabled resection, and is superior to single transmit 7T MRI.

**Key points**

- We scanned 31 patients with parallel transmit and conventional 7T magnetic resonance imaging (MRI), finding previously‐unreported structural lesions in nine patients (29% of cases).
- In 13% of cases, 7T MRI showed that an equivocal lesion at 3T MRI was likely significant.
- In 13% of cases, 7T MRI showed that an equivocal lesion at 3T MRI could be disregarded.
- Both qualitative and quantitative quality assessments indicate superiority of pTx images over circularly polarized (CP).
- Future clinical implementations of 7T MRI for epilepsy should utilize parallel transmit where possible.

## 1 INTRODUCTION

Magnetic resonance imaging (MRI) plays a crucial role in detecting structural lesions for presurgical planning in patients with drug‐resistant epilepsy.^1^ The odds of seizure freedom after epilepsy surgery are roughly doubled if a lesion can be visualized with MRI.^2^ The 7T Epilepsy Task Force's 2021 consensus paper recommends ultra‐high field (UHF) 7T MRI scanners for clinical use in this group.^3^ 7T MRI offers superior spatial resolution and sensitivity compared to traditional clinical 3T MRI.^4^, ^5^ It is important to note that 7T MRI can detect structural epileptogenic lesions that are not detectable by 3T MRI.^6^ Recent clinical results show superiority over 3T MRI,^7^, ^8^ and correspond well with histological findings.^9^

However, because of the physics of 7T MRI, images are susceptible to dark patches—signal dropouts in areas where the transmit B_1_^+^ field is weak. These dropouts commonly occur in the temporal lobes, which are regions of particular interest for epileptogenic lesion detection,^10^, ^11^, ^12^ as well as the cerebellum. Parallel transmit (pTx) MRI can substantially mitigate these dropouts, improving the uniformity of 7T MRI images to achieve diagnostic quality throughout the whole brain.^13^ A previous limitation of pTx was the need for time‐consuming per‐patient calibration scans, but recent developments in plug and play, template‐based universal pulses (UPs) allow much of the benefit of pTx at clinical timescales,^14^ at zero time penalty for the user and with no special expertise required.

In this study, we assess the feasibility and performance of pTx‐7T MRI for the clinical assessment of patients with drug‐resistant focal epilepsy using a protocol based on the recent 7T Epilepsy Task Force consensus recommendations.^3^ We implemented this in a “real‐world” epilepsy surgery pathway, scanning only those patients in whom conventional 3T MRI was inconclusive, and in whom further progress toward surgical resection would otherwise have either been impossible or required invasive stereotactic EEG (sEEG). We also acquired comparison images using conventional, circularly polarized (CP), 7T MRI.

## 2 METHODS

### 2.1 Patients

We recruited 32 consecutive adult patients with drug‐resistant focal epilepsy from the Cambridge Epilepsy Surgery Pathway. All had undergone inpatient videotelemetry (video‐EEG) in which seizures had been captured, and a fluorodeoxyglucose positron emission tomography (FDG‐PET) scan. All except two had 3T MRI imaging that was deemed negative or equivocal for the presence of a lesion; the remaining two had 3T‐visualied focal cortical dysplasia (FCD), but with unclear boundaries and potential impingement on dominant‐hemisphere language regions, and the epilepsy multi‐disciplinary team (MDT) requested 7T for better demarcation of extent. Two further patients (not included in the 32) were referred by the MDT but could not be recruited due to 7T contraindications (a tooth implant and an ear tattoo).

Patients were informed that imaging was being undertaken as part of a research study (National Research Ethics Service reference 23/WM/0008) and were informed that the scanner and sequences were not CE‐marked for clinical use. All provided informed consent to participate on this basis. Clinical re‐use of data as part of epilepsy surgery decision‐making was approved by Cambridge University Hospitals Governance and Legal structures and the UK national clinical negligence insurance scheme for trusts. A confirmation of non‐objection was obtained from the UK Medicines and Healthcare Regulatory Authority.

One of the patients could not be scanned due to large body habitus, so we report 31 datasets. This means that less than 10% of referred patients could not be scanned due to safety or practical barriers. The patients ranged from age 19 to age 60 (mean 34.8 years), with 15 being female and 16 male.

In four cases it was not possible to acquire the CP sequences for comparison, but in all cases we were able to complete sufficient sequences for clinical evaluation. Two patients were only able to tolerate part of the scan duration due to claustrophobia or having a seizure. Two patients ran out of scanner booking time due to arriving late and excessive motion requiring sequence repetition.

### 2.2 MRI acquisition

Data were acquired with a 7T MR system (MAGNETOM Terra, Siemens Healthcare, Erlangen, Germany) running VE12U SP01 software and equipped with an 8Tx/32Rx transmit/receive coil (Nova Medical, Wilmington, MA, USA). As shown in Table 1, we divided our imaging protocol into two parts. The first part comprised our candidate clinical package of parallel transmit 7T MRI sequences acquired with UP (PASTEUR package version 1.1): magnetization‐prepared 2 rapid acquisition gradient echo (MP2RAGE, producing both edge‐enhancing gradient echo [EDGE] contrast,^15^, ^16^ highlighting the gray‐white matter border, and UNI contrast, a traditional T_1_‐weighted image), fluid‐attenuated inversion recovery (FLAIR), and volumetric T_2_ turbo spin echo (TSE). To complete the 7T Epilepsy Task Force's recommended sequence list for clinical evaluation, we also acquired CP‐mode T_2_* and high‐resolution in‐plane 2D T_2_ TSE. PTx solutions for these sequences are in active development but were not available in time for the commencement of this study, so these sequences were used for clinical evaluation but did not form part of the pTx‐CP comparison. This clinical protocol took 40–45 min wall‐clock time in total (the total duration of the sequences was 35.5 min, but manual shimming was performed and patient comfort checked between sequences).

**TABLE 1:** Sequence parameters. Previous 7T magnetic resonance imaging (MRI) epilepsy studies have used conventional single transmit (sTx) 7T MRI with the Nova 1Tx32Rx head coil. For patient comfort and to minimize motion effects, we ran all scans using the Nova 8Tx32Rx head coil. This can be run in circularly polarized (CP) mode, also known as “TrueForm” mode, which is designed to closely emulate the performance of a conventional single transmit 7T MRI scan. We have previously shown that the 8Tx32Rx head coil has slightly better signal‐to‐noise ratio than the 1Tx32Rx head coil, so this choice will, if anything, underestimate the benefits of parallel transmit.

Sequence	Transmit mode	3D or 2D acquisition	Package	TR (ms)	TE (ms)	TI (ms)	Acquired voxel size (mm^3^)	Nominal FA (°)	FOV read (mm)	Slice thickness (mm)	Time (min)
**Proposed parallel transmit (pTx) 7T MRI clinical protocol**											
MP2RAGE	pTx	3D	PASTEUR^14^	3850	2.27	800/2700	0.8 × 0.8 × 0.8	5/7	240	.8	7:26
FLAIR	pTx	3D	PASTEUR	9200	326	2300	0.8 × 0.8 × 0.8	Variable	230	.8	6:54
T2 TSE	pTx	3D	PASTEUR	12 000	384	—	0.8 × 0.8 × 0.8	Variable	230	.8	8:00
**Control 7T scans in circularly polarized (CP) (or “TrueForm”) mode**											
T2 TSE	CP	2D	UK7T	8870	77	—	0.48 × 0.48 × 1	60	244	1	6:32
T2*	CP	3D	UK7T	31	20	—	0.8 × 0.8 × 0.8	15	224	.8	7:17
FLAIR	CP	3D	Siemens	9000	269	2600	0.8 × 0.8 × 0.8	variable	230	.8	7:23
MP2RAGE	CP	3D	Siemens	4300	1.99	840/2370	0.8 × 0.8 × 0.8	5/6	240	.8	8:33
**Parallel transmit research sequences to test feasibility in patients**											
DTI	pTx	2D	BOGAT	6400	65	—	0.8 × 0.8 × 1.25	90	210	1.25	3:57

Abbreviations: CP, circularly polarized; PTx, parallel transmit.

The second part comprised circularly polarized (CP, also called “TrueForm”) MP2RAGE and FLAIR scans for comparison, which are equivalent to conventional single transmit (sTx) 7T MRI. We acquired CP control scans rather than switching coils and rebooting the scanner for sTx acquisition to avoid confounding from between‐scan motion, and to make the study tolerable for the patients. These sequences took a further 16 min, resulting in ~1 h on the scanner in total.

For some subjects, if comfort and time permitted, we last performed a diffusion tensor imaging (DTI) scan with pTx Bayesian pulse optimization (BOGAT).^17^ This was for sequence development and evaluation purposes, and the results were not provided to the MDT and are not reported here.

All 3D sequences were acquired in a matched 0.8 mm isotropic spatial resolution and orientation to facilitate cross‐sequence comparison by the neuroradiology team. Full‐sequence protocol PDFs to support replication at other sites are provided in Appendix S1.

### 2.3 Clinical impact evaluation

Patients were referred for 7T MRI after the decision by the MDT that insufficient information was present to make a surgical proposal to the patient, and the next step would otherwise be sEEG, if a plausible hypothesis could be proposed, or consideration of non‐curative therapies such as vagus nerve stimulation if not.

After 7T imaging, patients were re‐discussed by the Cambridge MDT, and where necessary also at a joint meeting of the Cambridge and King's College Hospital MDTs (because sEEG for Cambridge patients is performed at King's, and they also provide quantitative, MRI‐coregistered PET analysis that was not available in Cambridge^18^). As part of this meeting, the presence or absence of contributory additional information from 7T‐MRI was agreed, and a consensus management decision made. A spreadsheet was kept that (1) recorded what additional information was provided by 7T‐MRI, if any, including whether a novel lesion had been demonstrated or an equivocal 3T finding clarified and (2) whether the 7T‐MRI changed clinical management, and if so in what way.

### 2.4 Image evaluation—qualitative

An MRI physicist (K.K.) created blind comparison images consisting of two sets of three matched orthogonal planes localized in the center of the brain, with a slight offset for sagittal slices to ensure that brain and not falx was included. Any patient in whom there was movement artifact in either the pTx or CP sequence was excluded from comparison. Comparison sets were selected randomly for each of the EDGE, T1‐UNI, and FLAIR sequences, with pTx and CP mode randomly assigned to be set “A” or set “B”, resulting in 42 paired judgments per rater. An example is provided in Figure S1.

The provision of single images rather than the whole dataset achieved three aims. The first was effective pTx vs CP blinding by order randomization. The second was that qualitative evaluation was based on overall image quality, rather than on the clarity of lesion demonstration, which was assessed separately. The third was to avoid raters making assessments based on signal dropout, as this was also assessed separately in the quantitative section. This is why central slices were chosen, where dropout was not usually evident, at least outside of inferior temporal regions on coronal section.

Image quality was assessed blindly by those members of the Cambridge MDT who would have clinical responsibility for their evaluation: two experienced neuroradiologists (J.P.J. and D.J.S.), the lead neurologist (T.E.C.), and lead neurosurgeon (R.M.). Raters were asked to score the images using the following 1–5 Likert scale: excellent diagnostic quality (5), good diagnostic quality (4), fair diagnostic quality (3), poor diagnostic quality (2), and non‐diagnostic quality (1).^19^

Quality ratings were statistically compared with a single repeated‐measures analysis of variance (ANOVA), implemented in Matlab 2020b, assessed for main effects of acquisition mode (pTx vs CP) and rater, as well as the within‐subject factor of sequence type (FLAIR vs EDGE vs UNI), plus the interaction of rater and acquisition mode. Post hoc paired *t* tests were used to explore significant ANOVA findings.

### 2.5 Image evaluation—quantitative

For a quantitative comparison of image uniformity we used Normalized Absolute Average Deviation (NAAD)^20^:

$$\text{NAAD}=100\times \left(1-\frac{1}{N\bar{Y}}\sum_{i=1}^{N}\left(\left|Y_{i}-\overset{̿}{Y}\right|\right)\right),$$

where: $Y_{i}$ is the individual pixel value in the region of interest (ROI), $\overset{̿}{Y}$ is the mean of all pixels in the ROI, and N is the total number of pixels in the ROI.

We calculated NAAD for white matter (WM) and gray matter (GM) separately, using the second inversion (INV2) images from MP2RAGE, as these have strong WM and GM signals that display nonuniformity if present (as opposed to INV1/EDGE, which are optimized to show the gray/white boundary, and UNI/T1, which are inhomogeneity corrected).

For brain segmentation we used FreeSurfer (v6.0.0)^21^ on UNI images from MP2RAGE acquisition. UNI images were co‐registered to atlas space and the transformation matrix was applied to the INV2 image using FLIRT from FSL package (v6.0.5). We extracted WM and GM from INV2 images and calculated NAAD for each separately.^20^

To determine the distribution of non‐uniformity across each acquisition method, we then plotted histograms of pixel intensity, and quantified deviation from a standardized normal distribution with the Wasserstein distance^22^ in Matlab 2020b.

## 3 RESULTS

### 3.1 Lesion demonstration

**FIGURE 1:**
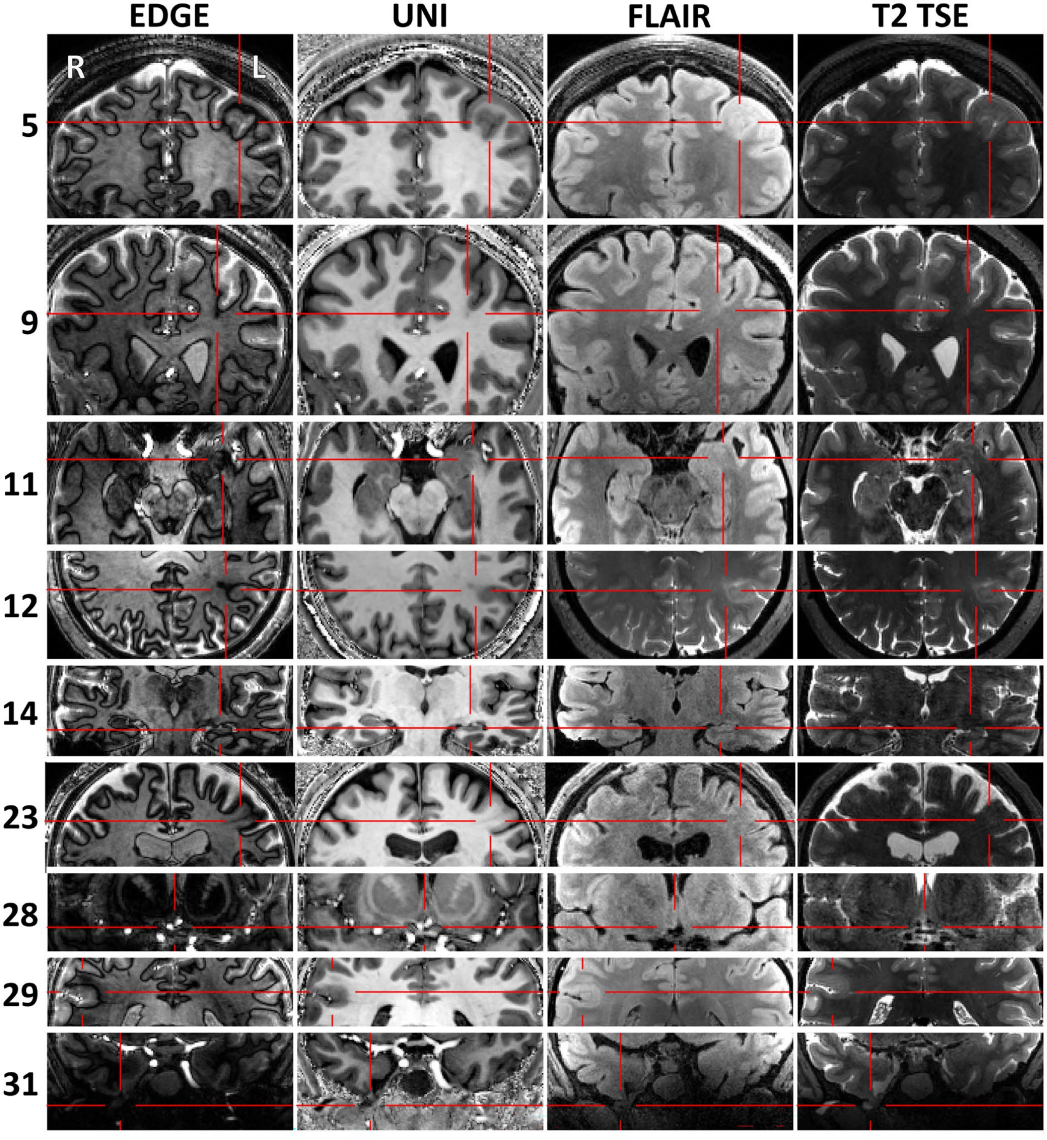
New lesions detected in the first 31 patients, deemed concordant and of high confidence by the MDT. Columns show the sequences. The identified lesions in Patient 5 (FCD), Patient 9 (FCD), Patient 11 (amygdala enlargement), Patient 12 (FCD), Patient 14 (hippocampal sclerosis), Patient 23 (FCD), Patient 28 (dysplasia or low grade glial/glioneural lesion), Patient 29 (FCD), Patient 31 (right temporal encephalocele).

**FIGURE 2:**
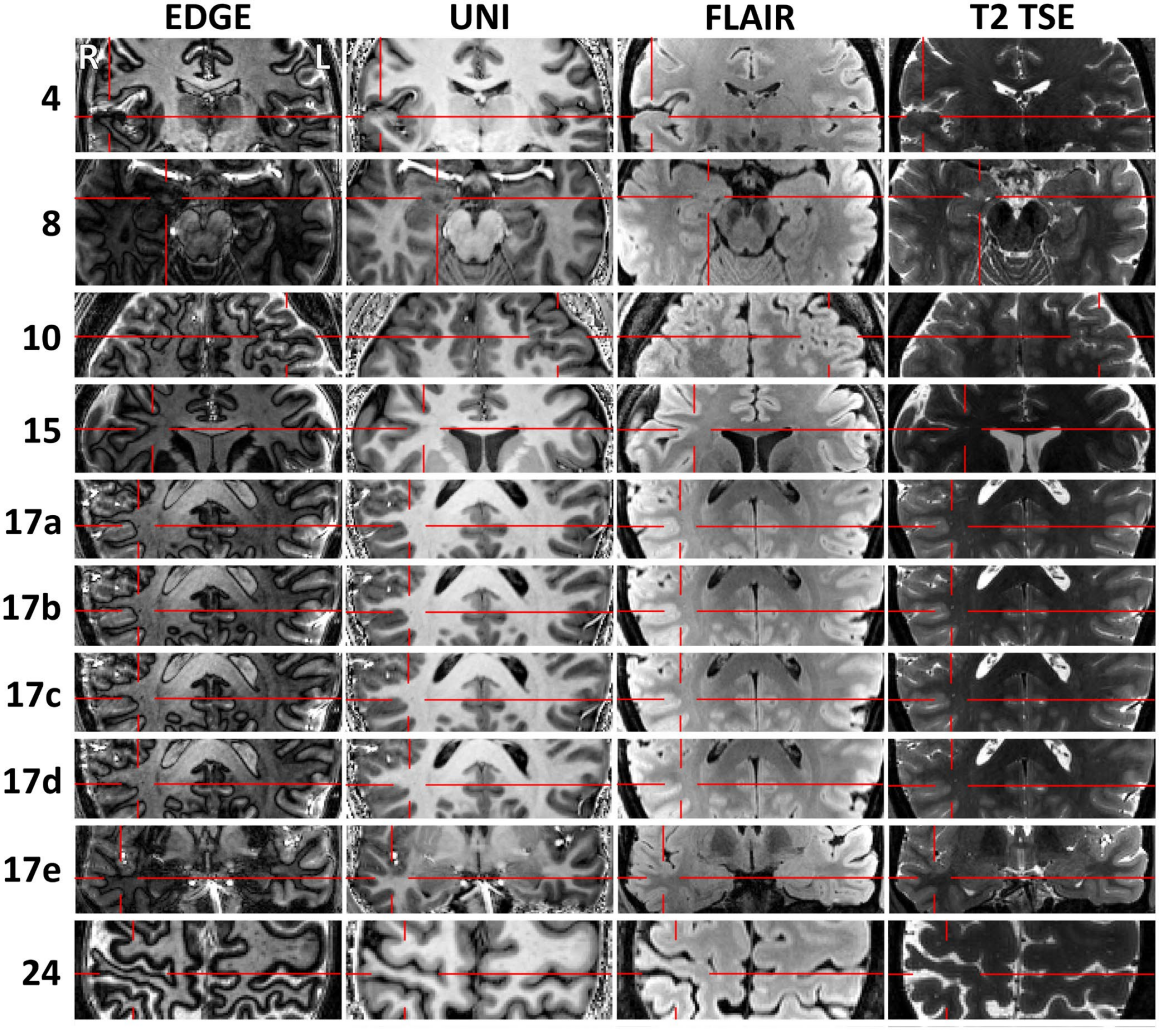
Putative lesions requiring further investigation before a surgical plan could be made. The putative lesions in: Patient 4 cortical–subcortical blurring of uncertain significance, stereo‐electroencephalography (sEEG) planned to evaluate significance; Patient 8 likely amygdala lesion, sEEG planned to evaluate significance; Patient 10 extensive polymicrogyria, sEEG planned to narrow down the epileptogenic zone; Patient 15 likely FCD, ictal SPECT requested for confirmation; Patient 17 rows a–d show four consecutive slices revealing that an equivocal lesion on 3T magnetic resonance imaging (MRI) is in fact a vessel, whereas row e shows a new possible FCD awaiting sEEG evaluation; Patient 24: Gray matter thinning likely representing perinatal infarction; sEEG planned to narrow down the epileptogenic zone.

7T revealed previously‐unseen structural lesions in nine patients (29%, Figure 1), confirmed 3T‐equivocal lesions in four patients (13%), and disproved 3T‐equivocal lesions in four patients (13%). Four patients had putative lesions detected requiring further investigation (Figure 2). Higher resolution images are in Appendix S2.

**FIGURE 3:**
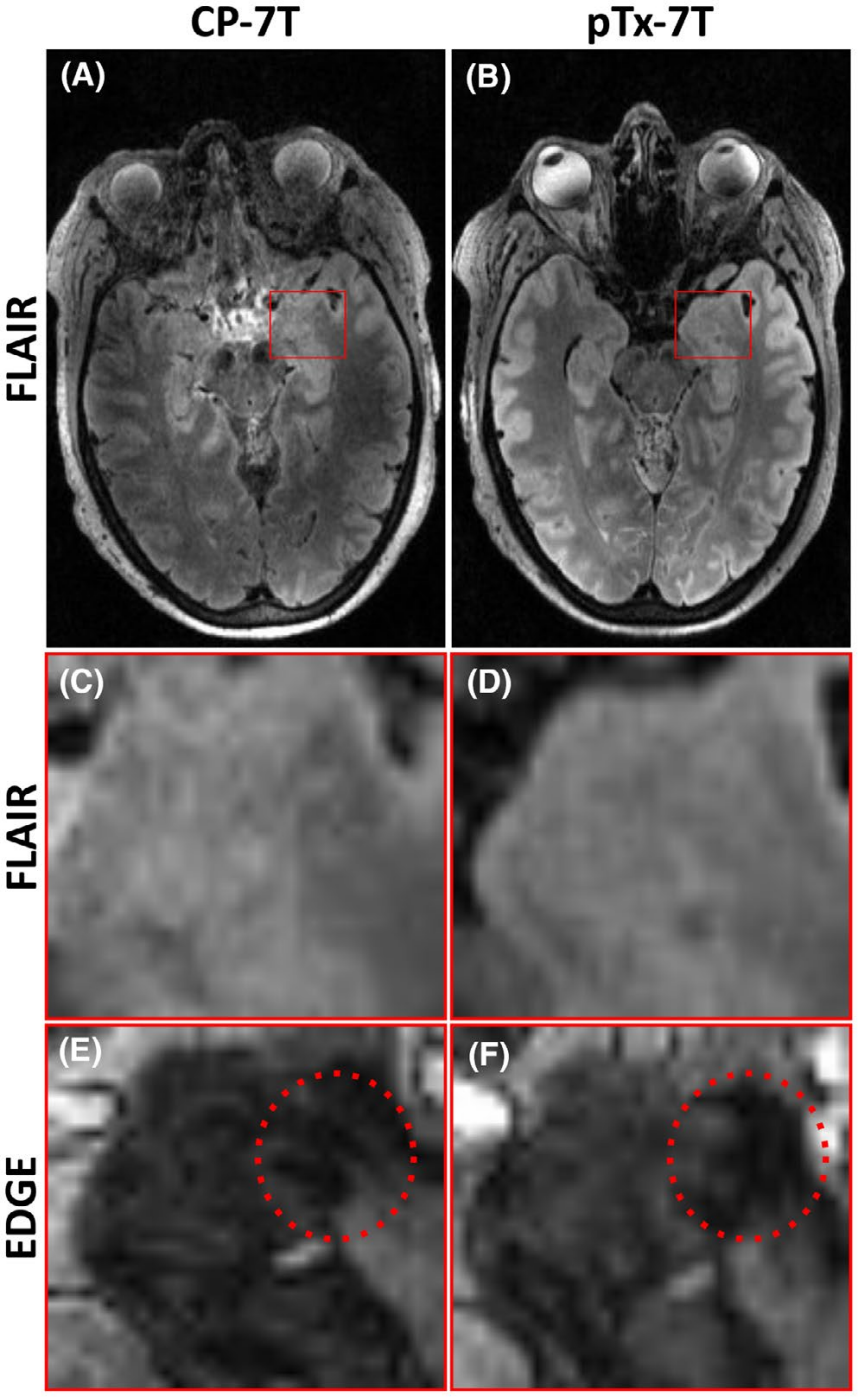
Patient 11 amygdala enlargement, visible on pTx but not CP images, due to improved uniformity of FLAIR sequence giving better definition of margins, and better clarity of EDGE contrast showing distension of lateral border.

**FIGURE 4:**
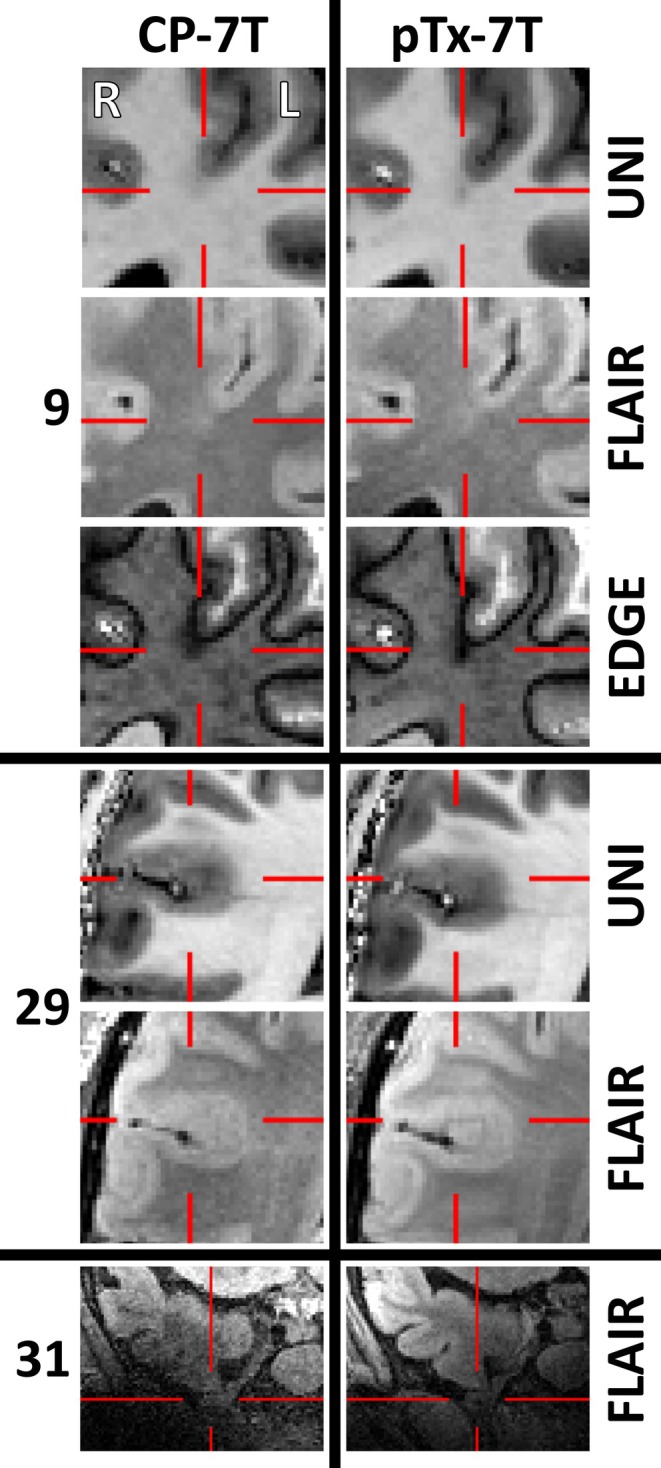
Further lesions better visualized with pTx than CP acquisitions. Patient 9: Focal cortical dysplasia much more prominently displayed and crisply defined, especially in the EDGE contrast. Patient 29: Focal cortical dysplasia not immediately evident on CP images, but clearly demonstrated with pTx. Patient 31: Inferior temporal encephalocele falling in an area of high signal dropout and low signal‐to‐noise ratio in CP FLAIR. Although the encephalocele can be seen with both acquisition methods it is impossible to assess the integrity of surrounding cortex in the CP images. MDT discussions often focus on whether an encephalocele may be associated with a more widespread cortical lesion and a more extensive resection may be warranted in addition to the skull‐base repair. This could be conducted with much greater confidence based on the pTx images compared to the CP images.

Subjective neuroradiology consensus was that new lesions were better visualized in pTx than CP mode for 6 patients, but in one case this was due to motion artifact in the CP acquisition, and in another the CP MP2RAGE acquisition could not be completed due to patient claustrophobia. We therefore conservatively report that four of seven (57%) of high‐confidence lesions were better visualized with pTx than CP acquisition (Figures 3 and 4), there was no difference in three of seven (43*%*), and lesions were never better visualized with CP.

### 3.2 Clinical value

In 18 of 31 patients (58%) the epilepsy MDT changed their clinical decision after having evaluated the 7T MRI images (note that this is greater than the 29% + 13% + 13% = 55% of patients where 7T changed the lesion demonstration [previous paragraph]. Patient 12 had a known lesion of unclear extent at 3T so does not contribute to lesion demonstration percentages, but did have a change in management as outlined in the vignettes). We illustrate the range and scope of this with clinical vignettes below. Overall, surgery was offered to nine patients in whom it would not otherwise have been possible without further investigation, and laser interstitial thermal therapy (LITT) to one patient. Three cases were removed from the surgical pathway because of bilateral or extensive lesions. Five cases were offered sEEG with better targeting (in three because the 7T lesion was deemed equivocal by the MDT, and in two because the lesion was extensive). One additional patient received a different operation, in part because 7T excluded a 3T‐equivocal lesion (this was confirmed by negative sEEG findings in the 3T‐implicated area, and after left temporal lobectomy the patient was found to have hippocampal sclerosis that was not visible at either 3T or 7T).

### 3.3 Image quality assessment—qualitative

A repeated‐measures ANOVA with Greenhouse Geisser correction confirmed a significant main effect of acquisition method (pTx vs CP; *F*(2, 184) = 13.7; *p* = 2.87 × 10^−6^) and a significant interaction between rater and acquisition method (*F*(2, 184) = 5.92; *p* = .00433). There was no significant main effect of rater (*F*(2, 184) = 1.52; *p* = .222), and a trend effect for sequence type (*F*(2, 184) = 2.81; *p* = .0679).

Post hoc tests demonstrated that this was because both neuroradiologists (*t*(11) = 4.75, *p* = 6.00 × 10^−4^; *t*(11) = 4.21, *p* = .0015) and the neurologist (*t*(11) = 4.69, *p* = 6.60 × 10^−4^) significantly preferred pTx FLAIR over CP FLAIR, whereas the neurosurgeon expressed no preference between the sequences. The neurosurgeon rated FLAIR image quality as very much higher overall (good or excellent diagnostic quality in every case for both acquisition methods), especially compared to the neuroradiologists, who provided average ratings of 2.33 and 2.42 (poor to fair diagnostic quality) for CP FLAIR and 3.75 and 3.33 (fair to good diagnostic quality) for pTx FLAIR. None of the raters displayed statistically significant rating differences for EDGE or UNI sequences (Table S1).

### 3.4 Image quality assessment—quantitative

**FIGURE 5:**
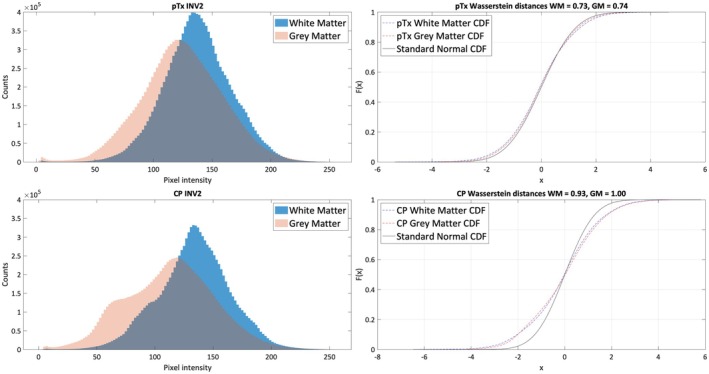
Left: Histograms of pixel intensity for the pTx and CP INV2 sequences. Right: Deviation of these distributions from a standard normal distribution, quantified by the Wasserstein distance—much higher for the CP than pTx sequences. CDF, cumulative distribution function.

Mean GM MP2RAGE INV2 NAAD of pTx images was 1.7% better (NAAD_GMpTx_ = 80.35%, NAAD_GMCP_ = 78.62%), equivalent to an 8% reduction in non‐uniformity, whereas WM was 0.46% better (NAAD_WMpTx_ = 86.17%, NAAD_WMCP_ = 85.71%), equivalent to a 3% reduction in non‐uniformity. Examination of the distribution of this non‐uniformity (Figure 5) demonstrated that pixel intensity for both WM and GM was much closer to a normal distribution for pTx (Wasserstein distances: WM = 0.73, GM = 0.74) than for CP (WM = 0.93, GM = 1.00). Specifically, CP images displayed a secondary peak of low pixel intensities, indicating signal drop‐out, which was near‐absent in the pTx images.

### 3.5 Case vignettes

#### 3.5.1 Patients where surgery was offered (Figure 1)

Patient 5, female, age 31. 7T demonstrates a left superior frontal cortex dysplasia, not visible at 3T, which was concordant with PET hypometabolism. The patient was offered surgery with awake craniotomy and electrocorticography for language, but this has been deferred due to improved seizure control.

Patient 9, female, age 26. 7T demonstrates focal cortical dysplasia of the left superior frontal sulcus with transmantle sign, concordant with PET. Previous 3T had demonstrated a malrotated hippocampus, but 7T demonstrated good hippocampal structure and no associated FCD. Surgery was offered.

Patient 11, female, age 55. 7T demonstrates new left amygdala enlargement, better visualized on pTx than CP sequences (Figure 3). Surgery was offered.

Patient 12, female, age 43. Patient already known to have left parietal FCD at 3T, but margins were poorly defined and there was potential impingement on language areas. 7T delineated the lesion very precisely. Surgery with awake craniotomy and electrocorticography for language was offered. Note that this patient was not included in our figures for lesion demonstration, because the lesion was already known from 3T, but was included in our figures for altered management as sEEG was no longer necessary.

Patient 14, male, age 43. 7T demonstrates new left hippocampal sclerosis, concordant with EEG. Surgery was performed, with histological confirmation of sclerosis in the resection specimen.

Patient 23, male, age 60. 3T demonstrates likely FCD in the frontal eye fields of unclear extent, confirmed, and much more precisely demonstrated at 7T. Surgery with intra‐operative functional mapping was offered.

Patient 28, male, age 23. 7T demonstrates a small lesion in the right paraterminal gyrus, likely representing dysplasia or a low grade glial/glioneuronal lesion. LITT was offered due to lesion size and deep location near the hypothalamus.

Patient 29, male, age 19. 3T equivocal right supramarginal gyrus dysplasia was confirmed and well demarcated at 7T. Surgery with intra‐operative functional mapping was offered.

Patient 31, female, age 22. 7T demonstrates right temporal meningocele, concordant with PET hypometabolism. Surgery was offered.

#### 3.5.2 Patients referred for targeted sEEG or other investigation (Figure 2)

Patient 4, female, age 37. 7T was reported initially as normal, but at MDT review of the right anterior mid‐temporal EEG onset was thought to have a potential correlate in cortical–subcortical blurring in the right superior temporal gyrus (STG)—patient listed for sEEG targeted there.

Patient 8, female, age 39. 7T demonstrates a likely right amygdala lesion. The patient was offered targeted sEEG because of bilateral EEG changes, negative PET, and non‐lateralizing semiology.

Patient 10, male, age 19. 7T demonstrates polymicrogyria in the left anterior frontal lobe with associated PET abnormality. Because the lesion is quite extensive, and close to language regions, sEEG was offered.

Patient 15, female, age 50. 3T demonstrated two equivocal lesions in the right frontal lobe. 7T recapitulated one lesion and excluded the other lesion. PET was normal, and there was much debate at MDT about whether sufficient information was present to offer surgery directly. Ultimately, out of caution, it was decided to perform ictal single photon emission computed tomography (SPECT)—if this is concordant, surgery will be offered, otherwise sEEG will be performed with better targeting.

Patient 17, female, age 20. 3T equivocal lesion in the right parietal lobe was demonstrated to be a vessel with thin cut 7T EDGE sequence. 7T demonstrates new equivocal lesion with PET concordance in the right superior temporal gyrus, but videotelemetry was discordant, so the patient was listed for targeted sEEG.

Patient 24, female, age 33. 7T demonstrates cortical thinning in the right post‐central gyrus, likely representing a peri‐natal infarction. Concordant with EEG and thought causative, but sEEG offered because of large lesion size and the potential for functional deficit.

#### 3.5.3 Surgical decision changed

Patient 1, female, age 24. Presumptive 3T lesion demonstrated to be venous spaces at 7T. Because of this, instead of proceeding directly to operative management the patient had sEEG. This confirmed an absence of abnormality in the 3T‐reported lesion, and instead implicated the left medial temporal lobe. A left temporal lobectomy was performed and histology confirmed hippocampal sclerosis. Even in retrospect, we do not think that this is evident at 3T or 7T, although quantitative analyses are in development.

Patient 6, male, age 48. 3T equivocal lesion in the right insula is demonstrated by 7T to be a peri‐vascular space. The patient was removed from the surgical pathway due to lack of a clear hypothesis for sEEG.

Patient 16, female, age 25. Patient was known to have right polymicrogyria at 3T, but 7T was requested because of some suspicion of the additional left hippocampal sclerosis. In fact, 7T demonstrated additional left‐sided polymicrogyria, so the patient was deemed ineligible for surgery and vagus nerve stimulation was offered instead.

## 4 DISCUSSION

This study tested the feasibility and impact of implementing a parallel transmit (or pTx) version of the 7T Epilepsy Task Force recommended 7T MRI protocols for epilepsy^3^ in a real‐world clinical epilepsy surgery pathway. We demonstrated that pTx‐7T influenced clinical management in 58% of cases, despite our patients having been selected because of negative or equivocal imaging at 3T. We directly evaluated the quality of pTx vs CP sequences and found pTx to be both qualitatively and quantitatively superior.

We demonstrated that pTx‐7T was feasible to implement. Less than 10% of patients were excluded due to 7T contraindications. No patients were unable to complete a clinically diagnostic set of sequences due to discomfort. Patient engagement activities undertaken after the study reported a high level of tolerability from patients, with only minor and occasional negative experiences such as dizziness on scanner entry and additional claustrophobia from the head coil. One patient experienced a seizure during their scan, but this was well managed by our radiography staff, who removed the patient from the scanner and made them safe, with high patient satisfaction afterwards.

Demonstration of new lesions in 29% of scanned patients, and clarification of 3T equivocal lesions in a further 26% (with a 50/50 split between verified and excluded) is more encouraging than a recent study by Hangel et al.,^12^ who implemented the task force consensus protocols in single transmit mode, finding new lesions in 19% of cases. This increased yield perhaps reflects our finding that four of seven high‐confidence lesions were better demonstrated in pTx than CP acquisitions.

In the majority of case series of epileptogenic lesions the most common abnormality is hippocampal sclerosis. Yet, in our study this represented only 1 of 13 detected lesions. There are two likely reasons for this. The first is that the incidence of hippocampal sclerosis has been observed to be decreasing over decades,^23^ likely as a result of lower incidences of childhood infection. The second is that hippocampal sclerosis is usually well detected at lower field strengths, and all of the patients in this study had negative or equivocal 3T MRI as an inclusion criterion. Although, with this in mind, it is interesting to note that our cohort included one patient who had hippocampal sclerosis in post‐operative histopathology that was not demonstrated, even in retrospect, at 3T or 7T.

Conversely, our study demonstrated a high proportion of malformations of cortical development, especially FCDs. These lesions are often small and subtle, being invisible or equivocal at lower field strengths. The high resolution of 7T acquisition, especially in combination with the EDGE contrast that emphasizes the gray/white matter border,^15^, ^16^ brings significant additional power to the demonstration of these epileptogenic lesions. Our subjective experience was that co‐registered FDG‐PET was particularly helpful in guiding the neuroradiological assessment, demonstrating concordance, and increasing lesion‐reporting confidence.

There was undoubtedly a learning curve over the course of this study, with neuroradiologists becoming more familiar with 7T images, and clinicians in the MDT becoming more confident in them with increasing experience. Naturally there was some initial skepticism about relying on a technique that does not yet have CE‐marking or multi‐center evaluation, and this may have led to some conservative decisions to proceed to additional investigation, especially for patients scanned early in the series. All of our clinical assessments relied on manual scan reads, informed at the MDT meeting by information from seizure semiology, video‐EEG, PET‐determined metabolism, and neuropsychological evaluation. These techniques are highly complementary, and 7T MRI forms only part of a multi‐modal evaluation seeking concordance, ensuring that surgery is performed only when the MDT has high confidence that the epileptogenic zone can be fully and safely resected. There is evidence that quantitative and automated image analysis methods can improve the sensitivity and confidence of lesion demonstration.^7^, ^24^, ^25^ These methods have not yet been validated in, or adapted to, pTx‐7T images, and this will be a focus of our future work.

Both pTx and CP images were available for clinical assessment. Our comparison images were acquired in CP mode, which uses the pTx coil to mimic sTx acquisition, but does not require coil swapping. We have shown previously that CP acquisition using Nova 8Tx head coil is equivalent to Nova 1Tx head coil acquisition, apart from the better signal‐to‐noise ratio (SNR) achieved with the former.^26^, ^27^ This means that any superiority of the pTx acquisition over CP is at least the same or better as the comparison with sTx acquisition using the standard Nova 1Tx head coil. There were four cases where pTx findings were qualitatively more informative to the epilepsy MDT than CP, and no cases where the reverse was true. This is a timely finding, since pTx‐7T systems became available as certified medical devices in 2023 and practically all new 7T sites will have access to these capabilities. When designing studies and clinical protocols for epilepsy our findings support centers utilizing pTx methods because we have shown that they already offer advantages. We expect this to improve further in the next few years as modern pTx scanners with VOP SAR (virtual observation point; specific absorption rate) supervision allow further innovation in pulse sequence design.

It is important to recognize that, although our pTx images were superior to CP in qualitative and quantitative image quality, they were not perfect, especially in the inferior temporal lobe. One patient with a 3T‐demonstrated inferior temporal meningocele, being scanned to ascertain whether there was an associated cortical dysplasia, had 7T images obscured by drop‐out in the relevant area with both pTx and CP. Similarly, the EDGE contrast frequently defined the GM/WM border less clearly in the temporal lobes than elsewhere. Work is ongoing to improve these issues. Similarly, we and others are developing pTx methods for 2D high‐resolution T_2_ acquisitions and susceptibility weighted imaging, to allow a full suite of pTx sequences for epilepsy evaluation. At present we recommend 7T MRI as next‐stage investigation only after 3T MRI, not yet as a direct replacement.

### 4.1 Limitations

Because of the delays inherent in performing neurosurgical resections and sEEG, the primary limitation of our study is that it reports only lesion visualization and MDT outcomes. More robust and objective outcomes are not yet available, but all of these patients will be followed up to more definitive endpoints in subsequent papers reporting sEEG‐7T concordance, histopathological data from resected 7T lesions, and ultimately patient seizure freedom and quality of life. However, we believe that our data are sufficient to justify that clinical 7T protocols should be established using parallel rather than single transmit.

Our study was only at a single center—we are currently developing a multi‐site extension.

We cannot exclude the possibility of bias, with clinicians involved in the study also being part of the MDT making decisions, although they always represented a minority of attendees and were very aware of their responsibilities to patient safety.

We compared the best available CP and pTx protocols at our institute, making minimal changes to meet the epilepsy task force recommendations and clinical considerations (most importantly using an identical 0.8 mm isotropic voxel size across each sequence). A consequence of this decision is that some scan parameters such as inversion time (TI) and bandwidth were not matched between pTx and CP acquisitions. The optimal values for these parameters are not known and may be different for pTx and CP scans because of differing inversion pulse efficiencies and magnetization transfer effects.^28^ Test acquisitions in which we matched parameters across acquisition types resulted in somewhat poorer image quality across all sequences (Figure S2).

Because the primary aim of our study was to assess the feasibility and utility of a pTx‐7T protocol for the evaluation of 3T‐negative epilepsy surgery patients we ensured that every patient had the best chance of obtaining a full set of pTx images by acquiring these first. Comparison CP images were acquired second, and were sacrificed in four patients (13%), who were not able to complete the entire scanning session. In general, scan participants are more likely to become uncomfortable and move at the end of scan sessions, so this may have unfairly disadvantaged the image quality of CP mode scans. We ameliorated this by explicitly excluding movement‐affected sequences from the qualitative and quantitative comparisons, but nonetheless this is a design compromise.

## 5 CONCLUSION

We demonstrate that the introduction of pTx‐7T MRI to a real‐world epilepsy surgery pathway changed clinical management for more than half of the patients scanned, making it highly likely that it will be cost‐effective in patients with normal 3T MRI, especially given its low cost in comparison to sEEG, and when considering the substantial health‐economic burdens of intractable epilepsy.

We show that pTx‐7T MRI is not inferior to sTx‐MRI in any case, and produces images of qualitatively and quantitatively superior quality. We therefore recommend that it should be adopted as the primary method for 7T MRI for epilepsy where possible.

## CONFLICT OF INTEREST STATEMENT

C.T.R. discloses research grant support from Siemens, for a different project. A.M. is employed by Siemens Healthcare SAS, Saint‐Denis, France. The remaining authors have no conflicts of interest. We confirm that we have read the Journal's position on issues involved in ethical publication and affirm that this report is consistent with those guidelines.

## Supporting information


Appendix S1.

Appendix S2.

Table S1.

Figure S1.

Figure S2.

## Data Availability

The data that support the findings of this study are available on request from the corresponding author. The data are not publicly available due to privacy or ethical restrictions.
