# Supplementary material for: Parallel transmit 7T MRI for adult epilepsy pre‐surgical evaluation
Source: Epilepsia. 2025 Mar 20;66(7):2315–27. doi: 10.1111/epi.18353 (PMC12291008; doi:10.1111/epi.18353)
Supplement: Supplementary file 1 — Appendix S1. [file EPI-66-2315-s004.pdf]

\\USER\WBIC Protocols Ready\Protocol 569\P00569_Epilepsy_20230628\AAHead_Scout_32ch-head-coil

TA: 0:18 PM: REF Voxel size: 1.6×1.6×1.6 mmPAT: 3 Rel. SNR: 1.00 : fl

Properties

Prio recon	Off
Load images to viewer	On
Inline movie	Off
Auto store images	On
Load images to stamp segments	Off
Load images to graphic segments	On
Auto open inline display	Off
Auto close inline display	Off
Start measurement without further preparation	Off
Wait for user to start	Off
Start measurements	Single measurement

Routine

Slab group	1
Slabs	1
Dist. factor	20 %
Position	L0.0 A0.5 H16.3 mm
Orientation	Sagittal
Phase enc. dir.	A >> P
Phase oversampling	0 %
Slice oversampling	0.0 %
Slices per slab	128
FoV read	260 mm
FoV phase	100.0 %
Slice thickness	1.6 mm
TR	4.17 ms
TE	1.53 ms
Averages	1
Concatenations	1
Filter	B1 filter
Coil elements	AC

Contrast - Common

TR	4.17 ms
TE	1.53 ms
Flip angle	16 deg

Contrast - Dynamic

Averages	1
Averaging mode	Short term
Reconstruction	Magnitude
Measurements	1

Resolution - Common

FoV read	260 mm
FoV phase	100.0 %
Slice thickness	1.6 mm
Base resolution	160
Phase resolution	100 %
Slice resolution	69 %
Phase partial Fourier	6/8
Slice partial Fourier	6/8
Trajectory	Cartesian

Resolution - iPAT

PAT mode	GRAPPA
Accel. factor PE	3
Ref. lines PE	24

Resolution - iPAT

Accel. factor 3D	1
Reference scan mode	Integrated

Resolution - Filter Image

Image Filter	Off
Distortion Corr.	Off
Prescan Normalize	Off
Normalize	Off
B1 filter	On
Unfiltered images	Off

Resolution - Filter Rawdata

Raw filter	Off
Elliptical filter	Off

Geometry - Common

Slab group	1
Slabs	1
Dist. factor	20 %
Position	L0.0 A0.5 H16.3 mm
Orientation	Sagittal
Phase enc. dir.	A >> P
Slice oversampling	0.0 %
Slices per slab	128
FoV read	260 mm
FoV phase	100.0 %
Slice thickness	1.6 mm
TR	4.17 ms
Multi-slice mode	Sequential
Series	Ascending
Concatenations	1

Geometry - AutoAlign

Slab group	1
Position	L0.0 A0.5 H16.3 mm
Orientation	Sagittal
Phase enc. dir.	A >> P
Initial Position	Isocenter
L	0.0 mm
P	0.0 mm
H	0.0 mm
Initial Rotation	0.00 deg
Initial Orientation	Transversal

Geometry - Tim Planning Suite

Set-n-Go Protocol	Off
Table position	H
Table position	0 mm
Inline Composing	Off

System - Miscellaneous

Positioning mode	REF
Table position	H
Table position	0 mm
MSMA	S - C - T
Sagittal	R >> L
Coronal	A >> P
Transversal	F >> H
Coil Combine Mode	Adaptive Combine

System - Miscellaneous

Save uncombined	Off
Matrix Optimization	Off
Coil Select Mode	Default

System - Adjustments

B0 Shim mode	Tune up
B1 Shim mode	TrueForm
Confirm freq. adjustment	Off
Assume Dominant Fat	Off
Assume Silicone	Off
Adjustment Tolerance	Auto

System - Adjust Volume

Position	Isocenter
Orientation	Transversal
Rotation	0.00 deg
A >> P	263 mm
R >> L	350 mm
F >> H	350 mm
Reset	Off

System - pTx Volumes

B1 Shim mode	TrueForm
Excitation	Non-sel.

System - Tx/Rx

Frequency 1H	297.231131 MHz
Correction factor	1
Gain	High
Img. Scale Cor.	1.000
Reset	Off
? Ref. amplitude 1H	0.000 V

Physio - PACE

Resp. control	Off
Concatenations	1

Inline - Common

Flip angle	16 deg
Measurements	1
Time to center	7.7 s

Inline - Inline

Subtract	Off
Measurements	1
StdDev	Off
Save original images	On

Inline - MIP

MIP-Sag	Off
MIP-Cor	Off
MIP-Tra	Off
MIP-Time	Off
Save original images	On

Inline - Composing

Inline Composing	Off
Distortion Corr.	Off

Inline - MapIt

Save original images	On
MapIt	None
Flip angle	16 deg

Inline - MapIt

Measurements	1
Contrasts	1
TR	4.17 ms
TE	1.53 ms

Sequence - Part 1

Introduction	On
Dimension	3D
Asymmetric echo	Weak
Contrasts	1
Multi-slice mode	Sequential
Bandwidth	540 Hz/Px

Sequence - Part 2

RF pulse type	Fast
Gradient mode	Normal
Excitation	Non-sel.
RF spoiling	On

Sequence - Assistant

Mode	Off

\\USER\WBIC Protocols Ready\Protocol 569\P00569_Epilepsy_20230628\flair_ns_tse_vfl_0.8iso_sag_UP

TA: 6:54 PM: FIX Voxel size: 0.8×0.8×0.8 mmPAT: 6 Rel. SNR: 1.00 : spcir

Properties

Prio recon	Off
Load images to viewer	On
Inline movie	Off
Auto store images	On
Load images to stamp segments	Off
Load images to graphic segments	Off
Auto open inline display	Off
Auto close inline display	Off
Start measurement without further preparation	Off
Wait for user to start	Off
Start measurements	Single measurement

Routine

Slab group	1
Slabs	1
Position	Isocenter
Orientation	Sagittal
Phase enc. dir.	A >> P
AutoAlign	---
Phase oversampling	0 %
Slice oversampling	0.0 %
Slices per slab	192
FoV read	230 mm
FoV phase	100.0 %
Slice thickness	0.80 mm
TR	9000 ms
TE	326 ms
Averages	1.0
Concatenations	1
Filter	Raw filter
Coil elements	AC

Contrast - Common

TR	9000 ms
TE	326 ms
MTC	Off
Magn. preparation	Non-sel. T2-IR
TI	2300 ms
T2prep duration	100 ms
Fat suppr.	None
Blood suppr.	Off
Restore magn.	Off

Contrast - Dynamic

Averages	1.0
Reconstruction	Magnitude
Measurements	1
Multiple series	Each measurement

Resolution - Common

FoV read	230 mm
FoV phase	100.0 %
Slice thickness	0.80 mm
Base resolution	288
Phase resolution	100 %
Slice resolution	100 %
Phase partial Fourier	Allowed
Slice partial Fourier	Off

Resolution - Common

Interpolation	Off

Resolution - iPAT

PAT mode	CAIPIRINHA
Accel. factor PE	3
Ref. lines PE	32
Accel. factor 3D	2
Ref. lines 3D	24
Reordering Shift 3D	1
Reference scan mode	Integrated

Resolution - Filter Image

Image Filter	Off
Distortion Corr.	Off
Prescan Normalize	Off
Normalize	Off
B1 filter	Off

Resolution - Filter Rawdata

Raw filter	On
Elliptical filter	Off

Geometry - Common

Slab group	1
Slabs	1
Position	Isocenter
Orientation	Sagittal
Phase enc. dir.	A >> P
Slice oversampling	0.0 %
Slices per slab	192
FoV read	230 mm
FoV phase	100.0 %
Slice thickness	0.80 mm
TR	9000 ms
Series	Ascending
Concatenations	1

Geometry - AutoAlign

Slab group	1
Position	Isocenter
Orientation	Sagittal
Phase enc. dir.	A >> P
AutoAlign	---
Initial Position	Isocenter
L	0.0 mm
P	0.0 mm
H	0.0 mm
Initial Rotation	0.00 deg
Initial Orientation	Sagittal

Geometry - Saturation

Fat suppr.	None
Restore magn.	Off
Special sat.	None

Geometry - Navigator

Geometry - Tim Planning Suite

Set-n-Go Protocol	Off
Table position	H
Table position	0 mm
Inline Composing	Off

System - Miscellaneous

Positioning mode	FIX
Table position	H
Table position	0 mm
MSMA	S - C - T
Sagittal	R >> L
Coronal	A >> P
Transversal	F >> H
Coil Combine Mode	Adaptive Combine
Save uncombined	Off
Matrix Optimization	Off
AutoAlign	---
Coil Select Mode	Default

System - Adjustments

B0 Shim mode	Brain
B1 Shim mode	TrueForm
Confirm freq. adjustment	Off
Assume Dominant Fat	Off
Assume Silicone	Off
Adjustment Tolerance	Auto

System - Adjust Volume

! Position	R0.9 P9.8 H15.0 mm
! Orientation	T > C-16.4 > S-1.3
! Rotation	2.64 deg
! A >> P	163 mm
! R >> L	160 mm
! F >> H	111 mm
Reset	Off

System - pTx Volumes

B1 Shim mode	TrueForm
Excitation	Non-sel.

System - Tx/Rx

Frequency 1H	297.231131 MHz
Correction factor	1
Gain	High
Img. Scale Cor.	1.000
Reset	Off
? Ref. amplitude 1H	0.000 V

Physio - Signal1

1st Signal/Mode	None
Trigger delay	0 ms
TR	9000 ms
Concatenations	1

Physio - Cardiac

Magn. preparation	Non-sel. T2-IR
TI	2300 ms
T2prep duration	100 ms
Fat suppr.	None
Dark blood	Off
FoV read	230 mm
FoV phase	100.0 %
Phase resolution	100 %

Physio - PACE

Resp. control	Off
Concatenations	1

Inline - Common

Subtract	Off
Measurements	1
StdDev	Off
Save original images	On

Inline - MIP

MIP-Sag	Off
MIP-Cor	Off
MIP-Tra	Off
MIP-Time	Off
Save original images	On

Inline - Composing

Inline Composing	Off
Distortion Corr.	Off

Sequence - Part 1

Introduction	Off
Dimension	3D
Elliptical scanning	Off
Reordering	Linear
Flow comp.	No
Echo spacing	5.48 ms
Bandwidth	294 Hz/Px

Sequence - Part 2

Echo train duration	915 ms
Gradient mode	Fast
Excitation	Non-sel.
Flip angle mode	T2 var
Turbo factor	218

Sequence - pTX Pulses

Universal Pulse	1
Pulse type	Excitation
Trajectory	External

Sequence - Assistant

Allowed delay	0 s

\\USER\WBIC Protocols Ready\Protocol 569\P00569_Epilepsy_20230628\Highresolution_TSE_PAT3_100_CTR1

TA: 6:32 PM: REF Voxel size: 0.5×0.5×1.0 mmPAT: 3 Rel. SNR: 1.00 : qtse

Properties

Prio recon	Off
Load images to viewer	On
Inline movie	Off
Auto store images	On
Load images to stamp segments	Off
Load images to graphic segments	Off
Auto open inline display	Off
Auto close inline display	Off
Start measurement without further preparation	Off
Wait for user to start	Off
Start measurements	Single measurement

Routine

Slice group	1
Slices	120
Dist. factor	10 %
Position	L0.0 P3.2 F8.5 mm
Orientation	C > T-14.7 > S0.1
Phase enc. dir.	R >> L
AutoAlign	---
Phase oversampling	0 %
FoV read	244 mm
FoV phase	100.0 %
Slice thickness	1.0 mm
TR	8870.0 ms
TE	77 ms
Averages	1
Concatenations	2
Filter	Raw filter
Coil elements	AC

Contrast - Common

TR	8870.0 ms
TE	77 ms
TD	0.0 ms
MTC	Off
Magn. preparation	None
Flip angle	60 deg
Fat suppr.	None
Water suppr.	None
Restore magn.	Off

Contrast - Dynamic

Averages	1
Averaging mode	Short term
Reconstruction	Magnitude
Measurements	1
Multiple series	Each measurement

Resolution - Common

FoV read	244 mm
FoV phase	100.0 %
Slice thickness	1.0 mm
Base resolution	512
Phase resolution	100 %
Phase partial Fourier	Off
Trajectory	Cartesian
Interpolation	Off

Resolution - iPAT

PAT mode	GRAPPA
Accel. factor PE	3
Ref. lines PE	27
Reference scan mode	Integrated

Resolution - Filter Image

Image Filter	Off
Distortion Corr.	Off
Prescan Normalize	Off
Normalize	Off
B1 filter	Off

Resolution - Filter Rawdata

Raw filter	On
Elliptical filter	Off

Geometry - Common

Slice group	1
Slices	120
Dist. factor	10 %
Position	L0.0 P3.2 F8.5 mm
Orientation	C > T-14.7 > S0.1
Phase enc. dir.	R >> L
FoV read	244 mm
FoV phase	100.0 %
Slice thickness	1.0 mm
TR	8870.0 ms
Multi-slice mode	Interleaved
Series	Interleaved
Concatenations	2

Geometry - AutoAlign

Slice group	1
Position	L0.0 P3.2 F8.5 mm
Orientation	C > T-14.7 > S0.1
Phase enc. dir.	R >> L
AutoAlign	---
Initial Position	L0.0 P3.2 F8.5
R	0.0 mm
P	3.2 mm
F	8.5 mm
Initial Rotation	-1.30 deg
Initial Orientation	C > T
C > T	-14.7
> S	0.1

Geometry - Saturation

Fat suppr.	None
Water suppr.	None
Restore magn.	Off
Special sat.	None

Geometry - Navigator

Geometry - Tim Planning Suite

Set-n-Go Protocol	Off
Table position	H
Table position	0 mm

Geometry - Tim Planning Suite

Inline Composing	Off

Geometry - Tim CT

Tim CT mode	Off
Slices	120
Slice thickness	1.0 mm
Dist. factor	10 %
FoV read	244 mm
FoV phase	100.0 %

System - Miscellaneous

Positioning mode	REF
Table position	F
Table position	0 mm
MSMA	S - C - T
Sagittal	R >> L
Coronal	A >> P
Transversal	F >> H
Coil Combine Mode	Sum of Squares
Save uncombined	Off
Matrix Optimization	Off
AutoAlign	---
Coil Select Mode	Default

System - Adjustments

B0 Shim mode	Brain
B1 Shim mode	TrueForm
Confirm freq. adjustment	Off
Assume Dominant Fat	Off
Assume Silicone	Off
Adjustment Tolerance	Auto

System - Adjust Volume

! Position	R0.9 P9.8 H15.0 mm
! Orientation	T > C-16.4 > S-1.3
! Rotation	2.64 deg
! A >> P	163 mm
! R >> L	160 mm
! F >> H	111 mm
Reset	Off

System - pTx Volumes

B1 Shim mode	TrueForm

System - Tx/Rx

Frequency 1H	297.231131 MHz
Correction factor	1
Gain	High
Img. Scale Cor.	1.000
Reset	Off
? Ref. amplitude 1H	0.000 V

Physio - Signal1

1st Signal/Mode	None
TR	8870.0 ms
Concatenations	2

Physio - Cardiac

Magn. preparation	None
Fat suppr.	None
Dark blood	Off
FoV read	244 mm
FoV phase	100.0 %

Physio - Cardiac

Phase resolution	100 %
Trajectory	Cartesian

Physio - PACE

Resp. control	Off
Concatenations	2

Inline - Common

Subtract	Off
Measurements	1
StdDev	Off
Save original images	On

Inline - MIP

MIP-Sag	Off
MIP-Cor	Off
MIP-Tra	Off
MIP-Time	Off
Save original images	On

Inline - Composing

Inline Composing	Off
Distortion Corr.	Off

Sequence - Part 1

Introduction	On
Dimension	2D
Compensate T2 decay	Off
Reduce Motion Sens.	Off
Contrasts	1
Flow comp.	No
Optimization	In phase
Multi-slice mode	Interleaved
Free echo spacing	On
Echo spacing	15.3 ms
Bandwidth	178 Hz/Px

Sequence - Part 2

Define	Turbo factor
Echo trains per slice	21
Phase correction	Automatic
Acoustic noise reduction	Active
RF pulse type	Low SAR
Gradient mode	Normal
Hyperecho	On
WARP	Off
Red. EC sensitivity	Off
Turbo factor	9

Sequence - Assistant

Mode	Off
Allowed delay	0 s

\\USER\WBIC Protocols Ready\Protocol 569\P00569_Epilepsy_20230628\t2_ns_tse_vfl_0.8iso_sag_UP

TA: 8:00 PM: FIX Voxel size: 0.8×0.8×0.8 mmPAT: 9 Rel. SNR: 1.00 : spc

Properties

Prio recon	Off
Load images to viewer	On
Inline movie	Off
Auto store images	On
Load images to stamp segments	Off
Load images to graphic segments	Off
Auto open inline display	Off
Auto close inline display	Off
Start measurement without further preparation	Off
Wait for user to start	Off
Start measurements	Single measurement

Routine

Slab group	1
Slabs	1
Position	Isocenter
Orientation	Sagittal
Phase enc. dir.	A >> P
AutoAlign	---
Phase oversampling	0 %
Slice oversampling	0.0 %
Slices per slab	240
FoV read	230 mm
FoV phase	100.0 %
Slice thickness	0.80 mm
TR	12000 ms
TE	384 ms
Averages	1.0
Concatenations	1
Filter	Raw filter
Coil elements	AC

Contrast - Common

TR	12000 ms
TE	384 ms
MTC	Off
Magn. preparation	None
Fat suppr.	None
Blood suppr.	Off
Restore magn.	Off

Contrast - Dynamic

Averages	1.0
Reconstruction	Magnitude
Measurements	1
Multiple series	Each measurement

Resolution - Common

FoV read	230 mm
FoV phase	100.0 %
Slice thickness	0.80 mm
Base resolution	288
Phase resolution	100 %
Slice resolution	100 %
Phase partial Fourier	Allowed
Slice partial Fourier	Off
Interpolation	Off

Resolution - iPAT

PAT mode	CAIPIRINHA
Accel. factor PE	3
Ref. lines PE	32
Accel. factor 3D	3
Ref. lines 3D	24
Reordering Shift 3D	1
Reference scan mode	Integrated

Resolution - Filter Image

Image Filter	Off
Distortion Corr.	Off
Prescan Normalize	Off
Normalize	Off
B1 filter	Off

Resolution - Filter Rawdata

Raw filter	On
Elliptical filter	Off

Geometry - Common

Slab group	1
Slabs	1
Position	Isocenter
Orientation	Sagittal
Phase enc. dir.	A >> P
Slice oversampling	0.0 %
Slices per slab	240
FoV read	230 mm
FoV phase	100.0 %
Slice thickness	0.80 mm
TR	12000 ms
Series	Ascending
Concatenations	1

Geometry - AutoAlign

Slab group	1
Position	Isocenter
Orientation	Sagittal
Phase enc. dir.	A >> P
AutoAlign	---
Initial Position	Isocenter
L	0.0 mm
P	0.0 mm
H	0.0 mm
Initial Rotation	0.00 deg
Initial Orientation	Sagittal

Geometry - Saturation

Fat suppr.	None
Restore magn.	Off
Special sat.	None

Geometry - Navigator

Geometry - Tim Planning Suite

Set-n-Go Protocol	Off
Table position	H
Table position	0 mm

Geometry - Tim Planning Suite

Inline Composing	Off

System - Miscellaneous

Positioning mode	FIX
Table position	H
Table position	0 mm
MSMA	S - C - T
Sagittal	R >> L
Coronal	A >> P
Transversal	F >> H
Coil Combine Mode	Adaptive Combine
Save uncombined	Off
Matrix Optimization	Off
AutoAlign	---
Coil Select Mode	Default

System - Adjustments

B0 Shim mode	Brain
B1 Shim mode	TrueForm
Confirm freq. adjustment	Off
Assume Dominant Fat	Off
Assume Silicone	Off
Adjustment Tolerance	Auto

System - Adjust Volume

! Position	R0.9 P9.8 H15.0 mm
! Orientation	T > C-16.4 > S-1.3
! Rotation	2.64 deg
! A >> P	163 mm
! R >> L	160 mm
! F >> H	111 mm
Reset	Off

System - pTx Volumes

B1 Shim mode	TrueForm
Excitation	Non-sel.

System - Tx/Rx

Frequency 1H	297.231131 MHz
Correction factor	1
Gain	High
Img. Scale Cor.	1.000
Reset	Off
? Ref. amplitude 1H	0.000 V

Physio - Signal1

1st Signal/Mode	None
Trigger delay	0 ms
TR	12000 ms
Concatenations	1

Physio - Cardiac

Magn. preparation	None
Fat suppr.	None
Dark blood	Off
FoV read	230 mm
FoV phase	100.0 %
Phase resolution	100 %

Physio - PACE

Resp. control	Off
Concatenations	1

Inline - Common

Subtract	Off
Measurements	1
StdDev	Off
Save original images	On

Inline - MIP

MIP-Sag	Off
MIP-Cor	Off
MIP-Tra	Off
MIP-Time	Off
Save original images	On

Inline - Composing

Inline Composing	Off
Distortion Corr.	Off

Sequence - Part 1

Introduction	Off
Dimension	3D
Elliptical scanning	Off
Reordering	Linear
Flow comp.	No
Echo spacing	3.52 ms
Bandwidth	694 Hz/Px

Sequence - Part 2

Echo train duration	764 ms
Gradient mode	Fast
Excitation	Non-sel.
Flip angle mode	T2 var
Turbo factor	218

Sequence - pTX Pulses

Universal Pulse	1
Pulse type	Excitation
Trajectory	External

Sequence - Assistant

Allowed delay	0 s

\\USER\WBIC Protocols Ready\Protocol 569\P00569_Epilepsy_20230628\T2star_0.8iso_GRAPPA_2_2

TA: 7:17 PM: FIX Voxel size: 0.8×0.8×0.8 mmPAT: 4 Rel. SNR: 1.00 : fl_r

Properties

Prio recon	Off
Load images to viewer	On
Inline movie	Off
Auto store images	On
Load images to stamp segments	Off
Load images to graphic segments	Off
Auto open inline display	Off
Auto close inline display	Off
Start measurement without further preparation	Off
Wait for user to start	Off
Start measurements	Single measurement

Routine

Slab group	1
Slabs	1
Dist. factor	20 %
Position	Isocenter
Orientation	Sagittal
Phase enc. dir.	A >> P
AutoAlign	---
Phase oversampling	0 %
Slice oversampling	0.0 %
Slices per slab	192
FoV read	224 mm
FoV phase	100.0 %
Slice thickness	0.80 mm
TR	31.0 ms
TE	20.00 ms
Averages	1
Concatenations	1
Filter	None
Coil elements	AC

Contrast - Common

TR	31.0 ms
TE	20.00 ms
MTC	Off
Magn. preparation	None
Flip angle	15 deg
Fat suppr.	None
Water suppr.	None
SWI	Off

Contrast - Dynamic

Averages	1
Averaging mode	Short term
Reconstruction	Magn./Phase
Measurements	1
Multiple series	Each measurement

Resolution - Common

FoV read	224 mm
FoV phase	100.0 %
Slice thickness	0.80 mm
Base resolution	280
Phase resolution	100 %
Slice resolution	100 %
Phase partial Fourier	Off

Resolution - Common

Slice partial Fourier	Off
Interpolation	Off

Resolution - iPAT

PAT mode	GRAPPA
Accel. factor PE	2
Ref. lines PE	40
Accel. factor 3D	2
Ref. lines 3D	24
Reference scan mode	Integrated

Resolution - Filter Image

Image Filter	Off
Distortion Corr.	Off
Prescan Normalize	Off
Normalize	Off
B1 filter	Off

Resolution - Filter Rawdata

Raw filter	Off
Elliptical filter	Off

Geometry - Common

Slab group	1
Slabs	1
Dist. factor	20 %
Position	Isocenter
Orientation	Sagittal
Phase enc. dir.	A >> P
Slice oversampling	0.0 %
Slices per slab	192
FoV read	224 mm
FoV phase	100.0 %
Slice thickness	0.80 mm
TR	31.0 ms
Multi-slice mode	Interleaved
Series	Interleaved
Concatenations	1

Geometry - AutoAlign

Slab group	1
Position	Isocenter
Orientation	Sagittal
Phase enc. dir.	A >> P
AutoAlign	---
Initial Position	Isocenter
L	0.0 mm
P	0.0 mm
H	0.0 mm
Initial Rotation	0.00 deg
Initial Orientation	Sagittal

Geometry - Saturation

Saturation mode	Standard
Fat suppr.	None
Water suppr.	None
Special sat.	None

Geometry - Tim Planning Suite

Set-n-Go Protocol	Off
Table position	H
Table position	0 mm
Inline Composing	Off

Geometry - Tim CT

Tim CT mode	Off
Slabs	1
Slices per slab	192
Slice thickness	0.80 mm
Dist. factor	20 %
FoV read	224 mm
FoV phase	100.0 %
Segments	1

System - Miscellaneous

Positioning mode	FIX
Table position	F
Table position	0 mm
MSMA	S - C - T
Sagittal	R >> L
Coronal	A >> P
Transversal	F >> H
Coil Combine Mode	Sum of Squares
Save uncombined	Off
Matrix Optimization	Off
AutoAlign	---
Coil Select Mode	Default

System - Adjustments

B0 Shim mode	Brain
B1 Shim mode	TrueForm
Confirm freq. adjustment	Off
Assume Dominant Fat	Off
Assume Silicone	Off
Adjustment Tolerance	None

System - Adjust Volume

! Position	R0.9 P9.8 H15.0 mm
! Orientation	T > C-16.4 > S-1.3
! Rotation	2.64 deg
! A >> P	163 mm
! R >> L	160 mm
! F >> H	111 mm
Reset	Off

System - pTx Volumes

B1 Shim mode	TrueForm
Excitation	Slab-sel.

System - Tx/Rx

Frequency 1H	297.231131 MHz
Correction factor	1
Gain	High
Img. Scale Cor.	1.000
Reset	Off
? Ref. amplitude 1H	0.000 V

Physio - Signal1

1st Signal/Mode	None
TR	31.0 ms
Concatenations	1
Segments	1

Physio - Cardiac

Tagging	None
Magn. preparation	None
Fat suppr.	None
Dark blood	Off
FoV read	224 mm
FoV phase	100.0 %
Phase resolution	100 %

Physio - PACE

Resp. control	Off
Concatenations	1

Inline - Common

Subtract	Off
Measurements	1
StdDev	Off
Liver registration	Off
Save original images	On

Inline - MIP

MIP-Sag	Off
MIP-Cor	Off
MIP-Tra	Off
MIP-Time	Off
Save original images	On

Inline - Soft Tissue

Wash - In	Off
Wash - Out	Off
TTP	Off
PEI	Off
MIP - time	Off
Measurements	1

Inline - Composing

Inline Composing	Off
Distortion Corr.	Off

Inline - MapIt

Save original images	On
MapIt	None
Flip angle	15 deg
Measurements	1
Contrasts	1
TR	31.0 ms
TE	20.00 ms

Sequence - Part 1

Introduction	On
Dimension	3D
Elliptical scanning	Off
Phase stabilisation	Off
Asymmetric echo	Off
Contrasts	1
Flow comp.	Slice/Read
Multi-slice mode	Interleaved
Bandwidth	80 Hz/Px

Sequence - Part 2

Segments	1
Acoustic noise reduction	None
RF pulse type	Normal
Gradient mode	Fast

Sequence - Part 2

Excitation	Slab-sel.
RF spoiling	On

Sequence - Assistant

Mode	Off

\\USER\WBIC Protocols Ready\Protocol 569\P00569_Epilepsy_20230628\dark-fluid_spcR_sag_p6_0.8

TA: 7:23 PM: FIX Voxel size: 0.8×0.8×0.8 mmPAT: 6 Rel. SNR: 1.00 : spcir

Properties

Prio recon	Off
Load images to viewer	On
Inline movie	Off
Auto store images	On
Load images to stamp segments	Off
Load images to graphic segments	Off
Auto open inline display	Off
Auto close inline display	Off
Start measurement without further preparation	Off
Wait for user to start	Off
Start measurements	Single measurement

Routine

Slab group	1
Slabs	1
Position	Isocenter
Orientation	Sagittal
Phase enc. dir.	A >> P
AutoAlign	---
Phase oversampling	0 %
Slice oversampling	8.3 %
Slices per slab	192
FoV read	230 mm
FoV phase	100.0 %
Slice thickness	0.80 mm
TR	9000 ms
TE	269 ms
Averages	1.0
Concatenations	1
Filter	B1 filter
Coil elements	AC

Contrast - Common

TR	9000 ms
TE	269 ms
MTC	Off
Magn. preparation	Non-sel. T2-IR
TI 1	2600 ms
Fat suppr.	None
Blood suppr.	Off
Restore magn.	Off

Contrast - Dynamic

Averages	1.0
Reconstruction	Magnitude
Measurements	1
Multiple series	Each measurement

Resolution - Common

FoV read	230 mm
FoV phase	100.0 %
Slice thickness	0.80 mm
Base resolution	288
Phase resolution	100 %
Slice resolution	100 %
Phase partial Fourier	Allowed
Slice partial Fourier	Off
Interpolation	Off

Resolution - iPAT

PAT mode	CAIPIRINHA
Accel. factor PE	3
Ref. lines PE	32
Accel. factor 3D	2
Ref. lines 3D	24
Reordering Shift 3D	1
Reference scan mode	Integrated

Resolution - Filter Image

Image Filter	Off
Distortion Corr.	Off
Prescan Normalize	Off
Normalize	Off
B1 filter	On
Unfiltered images	Off

Resolution - Filter Rawdata

Raw filter	Off
Elliptical filter	Off

Geometry - Common

Slab group	1
Slabs	1
Position	Isocenter
Orientation	Sagittal
Phase enc. dir.	A >> P
Slice oversampling	8.3 %
Slices per slab	192
FoV read	230 mm
FoV phase	100.0 %
Slice thickness	0.80 mm
TR	9000 ms
Series	Ascending
Concatenations	1

Geometry - AutoAlign

Slab group	1
Position	Isocenter
Orientation	Sagittal
Phase enc. dir.	A >> P
AutoAlign	---
Initial Position	Isocenter
L	0.0 mm
P	0.0 mm
H	0.0 mm
Initial Rotation	0.00 deg
Initial Orientation	Sagittal

Geometry - Saturation

Fat suppr.	None
Restore magn.	Off
Special sat.	None

Geometry - Navigator

Geometry - Tim Planning Suite

Set-n-Go Protocol	Off
Table position	H

Geometry - Tim Planning Suite

Table position	0 mm
Inline Composing	Off

System - Miscellaneous

Positioning mode	FIX
Table position	H
Table position	0 mm
MSMA	S - C - T
Sagittal	R >> L
Coronal	A >> P
Transversal	F >> H
Coil Combine Mode	Adaptive Combine
Save uncombined	Off
Matrix Optimization	Off
AutoAlign	---
Coil Select Mode	Default

System - Adjustments

B0 Shim mode	Brain
B1 Shim mode	TrueForm
Confirm freq. adjustment	Off
Assume Dominant Fat	Off
Assume Silicone	Off
Adjustment Tolerance	Auto

System - Adjust Volume

! Position	R0.9 P9.8 H15.0 mm
! Orientation	T > C-16.4 > S-1.3
! Rotation	2.64 deg
! A >> P	163 mm
! R >> L	160 mm
! F >> H	111 mm
Reset	Off

System - pTx Volumes

B1 Shim mode	TrueForm
Excitation	Non-sel.

System - Tx/Rx

Frequency 1H	297.231131 MHz
Correction factor	1
Gain	High
Img. Scale Cor.	5.000
Reset	Off
? Ref. amplitude 1H	0.000 V

Physio - Signal1

1st Signal/Mode	None
Trigger delay	0 ms
TR	9000 ms
Concatenations	1

Physio - Cardiac

Magn. preparation	Non-sel. T2-IR
TI 1	2600 ms
Fat suppr.	None
Dark blood	Off
FoV read	230 mm
FoV phase	100.0 %
Phase resolution	100 %

Physio - PACE

Resp. control	Off

Physio - PACE

Concatenations	1

Inline - Common

Subtract	Off
Measurements	1
StdDev	Off
Save original images	On

Inline - MIP

MIP-Sag	Off
MIP-Cor	Off
MIP-Tra	Off
MIP-Time	Off
Save original images	On

Inline - Composing

Inline Composing	Off
Distortion Corr.	Off

Sequence - Part 1

Introduction	On
Dimension	3D
Elliptical scanning	Off
Reordering	Linear
Flow comp.	No
Echo spacing	3.32 ms
Adiabatic-mode	Off
Bandwidth	723 Hz/Px

Sequence - Part 2

Echo train duration	631 ms
RF pulse type	Normal
Gradient mode	Fast
Excitation	Non-sel.
Flip angle mode	T2 var
Turbo factor	220

Sequence - Assistant

Allowed delay	120 s

\\USER\WBIC Protocols Ready\Protocol 569\P00569_Epilepsy_20230628\mp2rage_sag_p3_0.8mm
TA: 8:33 PM: REF Voxel size: 0.8×0.8×0.8 mmPAT: 3 Rel. SNR: 1.00 : tfl

Properties

Prio recon	Off
Load images to viewer	On
Inline movie	Off
Auto store images	On
Load images to stamp segments	Off
Load images to graphic segments	Off
Auto open inline display	Off
Auto close inline display	Off
Start measurement without further preparation	Off
Wait for user to start	Off
Start measurements	Single measurement

Routine

Slab group	1
Slabs	1
Dist. factor	50 %
Position	Isocenter
Orientation	Sagittal
Phase enc. dir.	A >> P
AutoAlign	---
Phase oversampling	0 %
Slice oversampling	0.0 %
Slices per slab	192
FoV read	240 mm
FoV phase	100.0 %
Slice thickness	0.80 mm
TR	4300.0 ms
TE	1.99 ms
Averages	1
Concatenations	1
Filter	None
Coil elements	AC

Contrast - Common

TR	4300.0 ms
TE	1.99 ms
Magn. preparation	Non-sel. IR
TI 1	840 ms
TI 2	2370 ms
Flip angle 1	5.0 deg
Flip angle 2	6.0 deg
Fat suppr.	Water excit. fast
Water suppr.	None

Contrast - Dynamic

Averages	1
Averaging mode	Long term
Reconstruction	Magnitude
Measurements	1
Multiple series	Each measurement

Resolution - Common

FoV read	240 mm
FoV phase	100.0 %
Slice thickness	0.80 mm
Base resolution	288
Phase resolution	100 %
Slice resolution	100 %
Phase partial Fourier	Off

Resolution - Common

Slice partial Fourier	6/8
Interpolation	Off

Resolution - iPAT

PAT mode	GRAPPA
Accel. factor PE	3
Ref. lines PE	36
Accel. factor 3D	1
Reference scan mode	Integrated

Resolution - Filter Image

Image Filter	Off
Distortion Corr.	Off
Prescan Normalize	Off
Normalize	Off
B1 filter	Off

Resolution - Filter Rawdata

Raw filter	Off
Elliptical filter	Off

Geometry - Common

Slab group	1
Slabs	1
Dist. factor	50 %
Position	Isocenter
Orientation	Sagittal
Phase enc. dir.	A >> P
Slice oversampling	0.0 %
Slices per slab	192
FoV read	240 mm
FoV phase	100.0 %
Slice thickness	0.80 mm
TR	4300.0 ms
Multi-slice mode	Single shot
Series	Interleaved
Concatenations	1

Geometry - AutoAlign

Slab group	1
Position	Isocenter
Orientation	Sagittal
Phase enc. dir.	A >> P
AutoAlign	---
Initial Position	Isocenter
L	0.0 mm
P	0.0 mm
H	0.0 mm
Initial Rotation	21.00 deg
Initial Orientation	Sagittal

Geometry - Navigator

Geometry - Tim Planning Suite

Set-n-Go Protocol	Off
Table position	H
Table position	0 mm
Inline Composing	Off

System - Miscellaneous

Positioning mode	REF
Table position	H
Table position	0 mm
MSMA	S - C - T
Sagittal	R >> L
Coronal	A >> P
Transversal	F >> H
Coil Combine Mode	Sum of Squares
Save uncombined	Off
Matrix Optimization	Off
AutoAlign	---
Coil Select Mode	Default

System - Adjustments

B0 Shim mode	Brain
B1 Shim mode	TrueForm
Confirm freq. adjustment	Off
Assume Dominant Fat	Off
Assume Silicone	Off
Adjustment Tolerance	Auto

System - Adjust Volume

! Position	R0.9 P9.8 H15.0 mm
! Orientation	T > C-16.4 > S-1.3
! Rotation	2.64 deg
! A >> P	163 mm
! R >> L	160 mm
! F >> H	111 mm
Reset	Off

System - pTx Volumes

B1 Shim mode	TrueForm
Excitation	Non-sel.

System - Tx/Rx

Frequency 1H	297.231131 MHz
Correction factor	1
Gain	High
Img. Scale Cor.	1.000
Reset	Off
? Ref. amplitude 1H	0.000 V

Physio - Signal1

1st Signal/Mode	None
TR	4300.0 ms
Concatenations	1

Physio - Cardiac

Magn. preparation	Non-sel. IR
TI 1	840 ms
TI 2	2370 ms
Fat suppr.	Water excit. fast
Dark blood	Off
FoV read	240 mm
FoV phase	100.0 %
Phase resolution	100 %

Physio - PACE

Resp. control	Off
Concatenations	1

Inline - Common

Subtract	Off

Inline - Common

Measurements	1
StdDev	Off
Save original images	On

Inline - MIP

MIP-Sag	Off
MIP-Cor	Off
MIP-Tra	Off
MIP-Time	Off
Save original images	On

Inline - Composing

Inline Composing	Off
Distortion Corr.	Off

Inline - MapIt

Save original images	On
MapIt	None
Flip angle 1	5.0 deg
Flip angle 2	6.0 deg
Measurements	1
TR	4300.0 ms
TE	1.99 ms

Sequence - Part 1

Introduction	On
Dimension	3D
Elliptical scanning	Off
Reordering	Linear
Asymmetric echo	Allowed
Flow comp.	No
Multi-slice mode	Single shot
Echo spacing	6.3 ms
Bandwidth	250 Hz/Px

Sequence - Part 2

RF pulse type	Fast
Gradient mode	Fast*
Excitation	Non-sel.
RF spoiling	On
Incr. Gradient spoiling	Off
Turbo factor	144

Sequence - Assistant

Mode	Off

\\USER\WBIC Protocols Ready\Protocol 569\P00569_Epilepsy_20230628\extra_ep2d_diff_PTX

TA: 3:57 PM: FIX Voxel size: 1.3×1.3×1.3 mmPAT: 3 Rel. SNR: 1.00 : epse

Properties

Prio recon	Off
Load images to viewer	On
Inline movie	Off
Auto store images	On
Load images to stamp segments	Off
Load images to graphic segments	Off
Auto open inline display	Off
Auto close inline display	Off
Start measurement without further preparation	Off
Wait for user to start	Off
Start measurements	Single measurement

Routine

Slice group	1
Slices	60
Dist. factor	0 %
Position	L0.0 P0.6 H7.3 mm
Orientation	Transversal
Phase enc. dir.	A >> P
AutoAlign	---
Phase oversampling	0 %
FoV read	210 mm
FoV phase	100.0 %
Slice thickness	1.25 mm
TR	6400 ms
TE	65.0 ms
Concatenations	1
Filter	None
Coil elements	AC

Contrast - Common

TR	6400 ms
TE	65.0 ms
MTC	Off
Magn. preparation	None
Flip angle exc	90 deg
Flip angle fat sat	110 deg
Fat suppr.	Fat sat.
Fat sat. mode	Weak

Contrast - Dynamic

Averaging mode	Long term
Reconstruction	Magnitude
Measurements	1
Delay in TR	0 ms
Multiple series	Off

Resolution - Common

FoV read	210 mm
FoV phase	100.0 %
Slice thickness	1.25 mm
Base resolution	168
Phase resolution	100 %
Phase partial Fourier	6/8
Interpolation	Off

Resolution - iPAT

Accel. mode	GRAPPA
Accel. factor PE	3

Resolution - iPAT

Ref. lines PE	66
Reference scan mode	GRE/separate

Resolution - Filter Image

Distortion Corr.	Off
Prescan Normalize	Off
Dynamic Field Corr.	Off

Resolution - Filter Rawdata

Raw filter	Off
Elliptical filter	Off

Geometry - Common

Slice group	1
Slices	60
Dist. factor	0 %
Position	L0.0 P0.6 H7.3 mm
Orientation	Transversal
Phase enc. dir.	A >> P
FoV read	210 mm
FoV phase	100.0 %
Slice thickness	1.25 mm
TR	6400 ms
Multi-slice mode	Interleaved
Series	Interleaved
Concatenations	1

Geometry - AutoAlign

Slice group	1
Position	L0.0 P0.6 H7.3 mm
Orientation	Transversal
Phase enc. dir.	A >> P
AutoAlign	---
Initial Position	L0.0 P0.6 H7.3
L	0.0 mm
P	0.6 mm
H	7.3 mm
Initial Rotation	0.00 deg
Initial Orientation	Transversal

Geometry - Saturation

Fat suppr.	Fat sat.
Fat sat. mode	Weak
Special sat.	None

Geometry - Navigator

Geometry - Tim Planning Suite

Set-n-Go Protocol	Off
Table position	H
Table position	0 mm
Inline Composing	Off

System - Miscellaneous

Positioning mode	FIX
Table position	H
Table position	0 mm
MSMA	S - C - T
Sagittal	R >> L

System - Miscellaneous

Coronal	A >> P
Transversal	F >> H
Coil Combine Mode	Sum of Squares
Matrix Optimization	Off
AutoAlign	---
Coil Select Mode	Default

System - Adjustments

B0 Shim mode	Brain
B1 Shim mode	TrueForm
Confirm freq. adjustment	Off
Assume Dominant Fat	Off
Assume Silicone	Off
Adjustment Tolerance	Auto

System - Adjust Volume

! Position	R0.9 P9.8 H15.0 mm
! Orientation	T > C-16.4 > S-1.3
! Rotation	2.64 deg
! A >> P	163 mm
! R >> L	160 mm
! F >> H	111 mm
Reset	Off

System - pTx Volumes

B1 Shim mode	TrueForm
Excitation	Standard

System - Tx/Rx

Frequency 1H	297.231131 MHz
Correction factor	1
Gain	High
Img. Scale Cor.	1.000
Reset	Off
? Ref. amplitude 1H	0.000 V

Physio - Signal1

1st Signal/Mode	None
TR	6400 ms
Concatenations	1

Physio - PACE

Resp. control	Off
Concatenations	1

Diff - Neuro

Diffusion mode	Free
Diff. directions	30
Diffusion Scheme	Monopolar
Diff. weightings	2
b-value 1	0 s/mm²
b-value 2	1000 s/mm²
b-value 1	2
b-value 2	1
Diff. weighted images	On
Trace weighted images	On
ADC maps	On
FA maps	On
Mosaic	Off
Tensor	On
Noise level	40

Diff - Body

Diffusion mode	Free
Diff. directions	30
Diffusion Scheme	Monopolar
Diff. weightings	2
b-value 1	0 s/mm²
b-value 2	1000 s/mm²
b-value 1	2
b-value 2	1
Diff. weighted images	On
Trace weighted images	On
ADC maps	On
Exponential ADC Maps	Off
FA maps	On
Invert Gray Scale	Off
Calculated Image	Off
b-Value >=	0 s/mm²
Noise level	40

Diff - Composing

Inline Composing	Off
Distortion Corr.	Off

Sequence - Part 1

Introduction	On
Optimization	None
Multi-slice mode	Interleaved
Free echo spacing	Off
Echo spacing	0.81 ms
Bandwidth	1418 Hz/Px

Sequence - Part 2

EPI factor	168
RF pulse type	Low SAR
Gradient mode	Normal
Excitation	Standard

Sequence - pTX Pulses

Sequence - Special

Use ptx pulse	On
Exc. pul. dur	10300 us
Refoc. pul. dur	11900 us
Exc. pul. phase	90 deg
Refoc. pul. phase	0 deg
Exc. pul. TE con.	3120 us
Refoc. pul. TE con.	2560 us
n(exc. pulses)	1
n(refoc. pulses)	1
