# Supplementary material for: Parallel transmit 7T MRI for adult epilepsy pre‐surgical evaluation
Source: Epilepsia. 2025 Mar 20;66(7):2315–27. doi: 10.1111/epi.18353 (PMC12291008; doi:10.1111/epi.18353)
Supplement: Supplementary file 2 — Appendix S2. [file EPI-66-2315-s002.pdf]

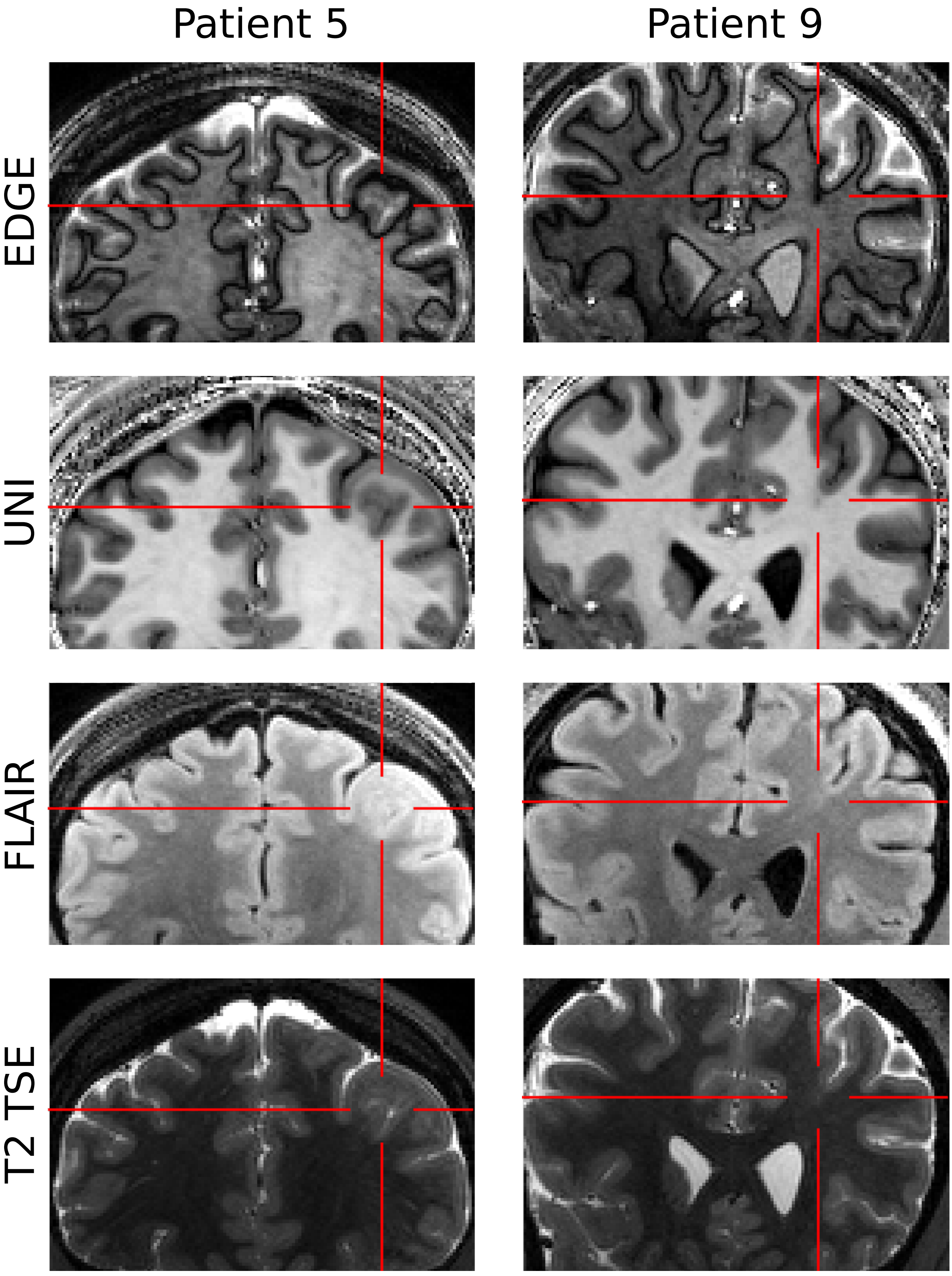

SI 4. High resolution images from Figure 2 part 1.

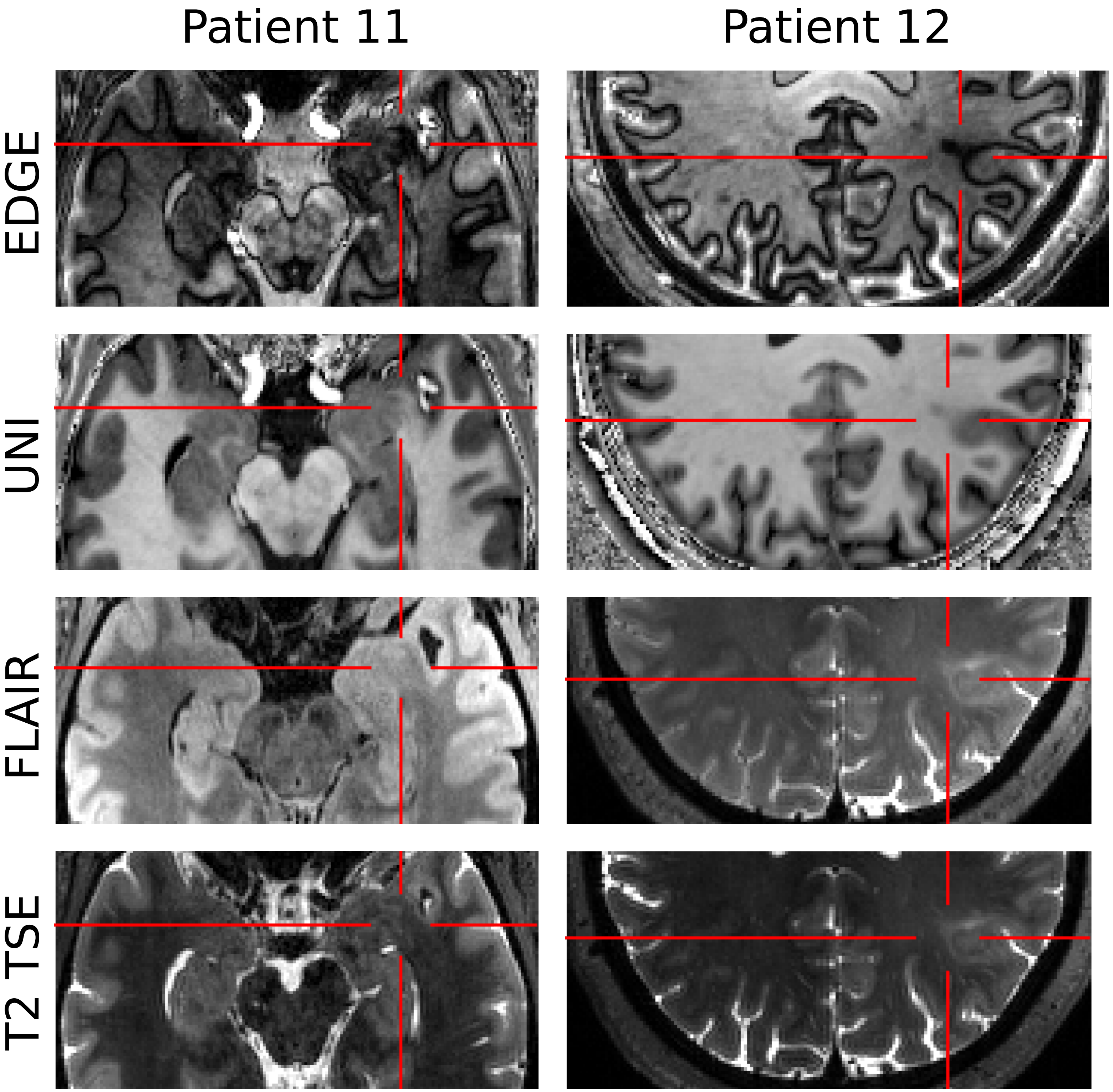

SI 5. High resolution images from Figure 2 part 2.

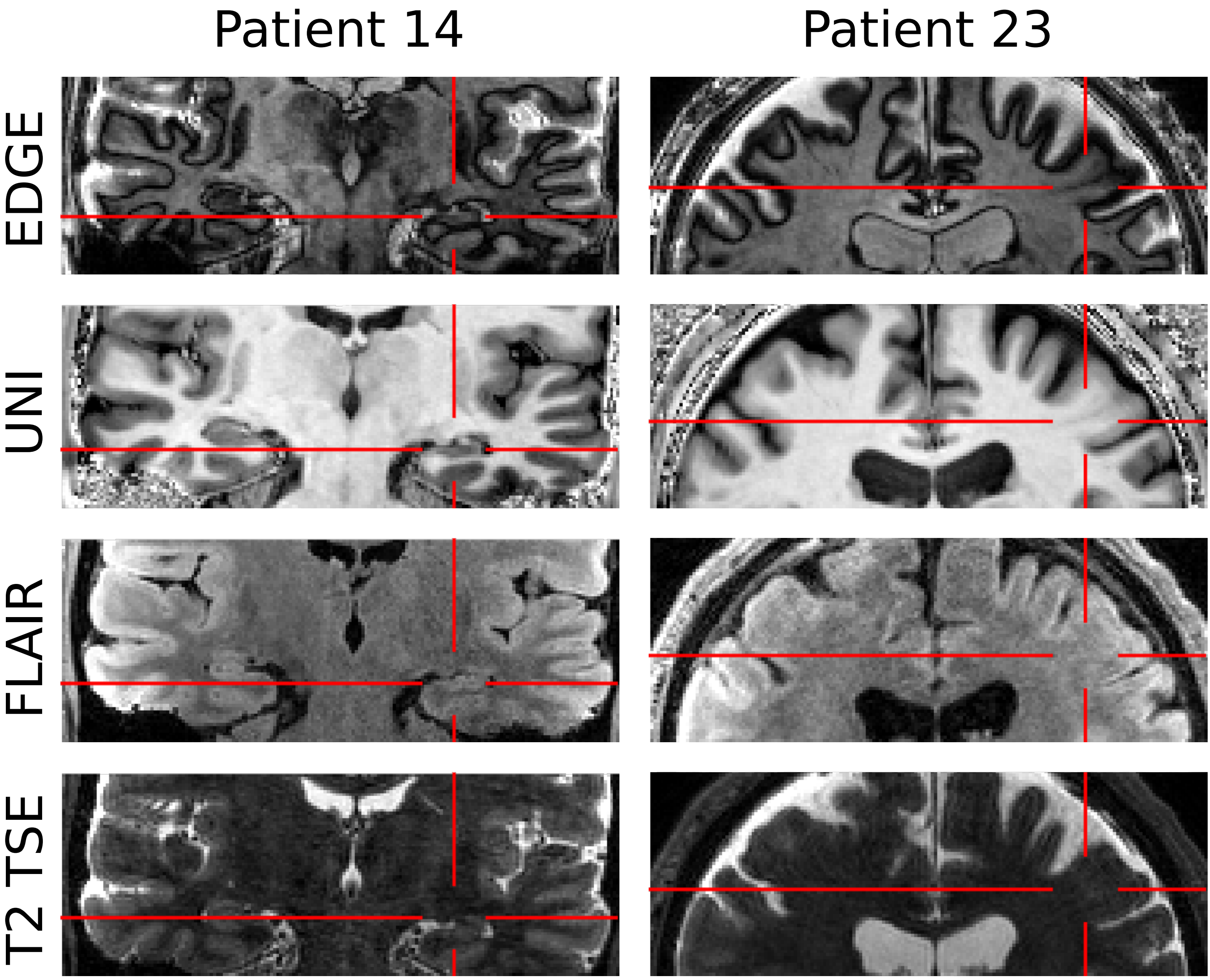

SI 6. High resolution images from Figure 2 part 3.

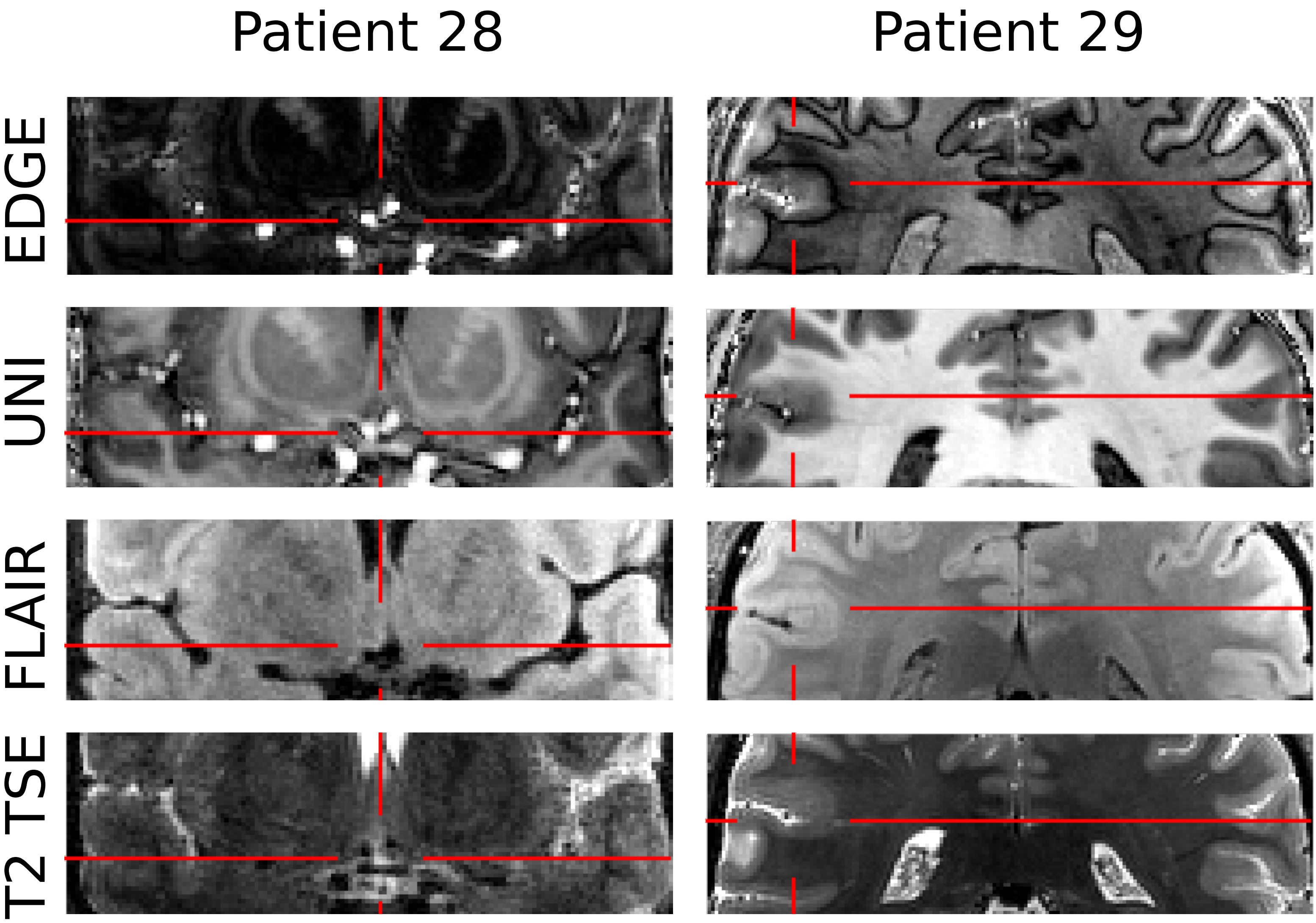

SI 7. High resolution images from Figure 2 part 4.

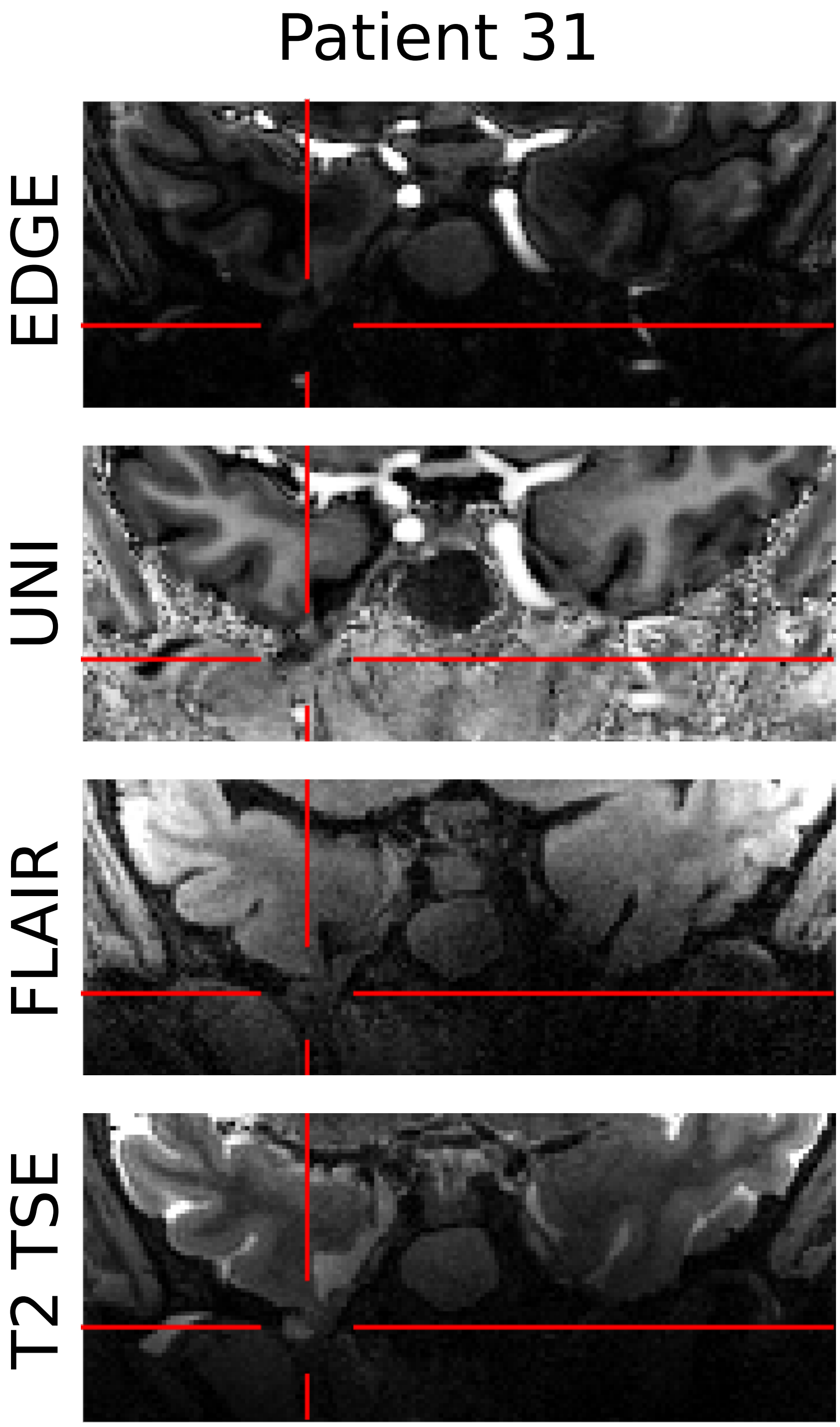

SI 8. High resolution images from Figure 2 part 5.

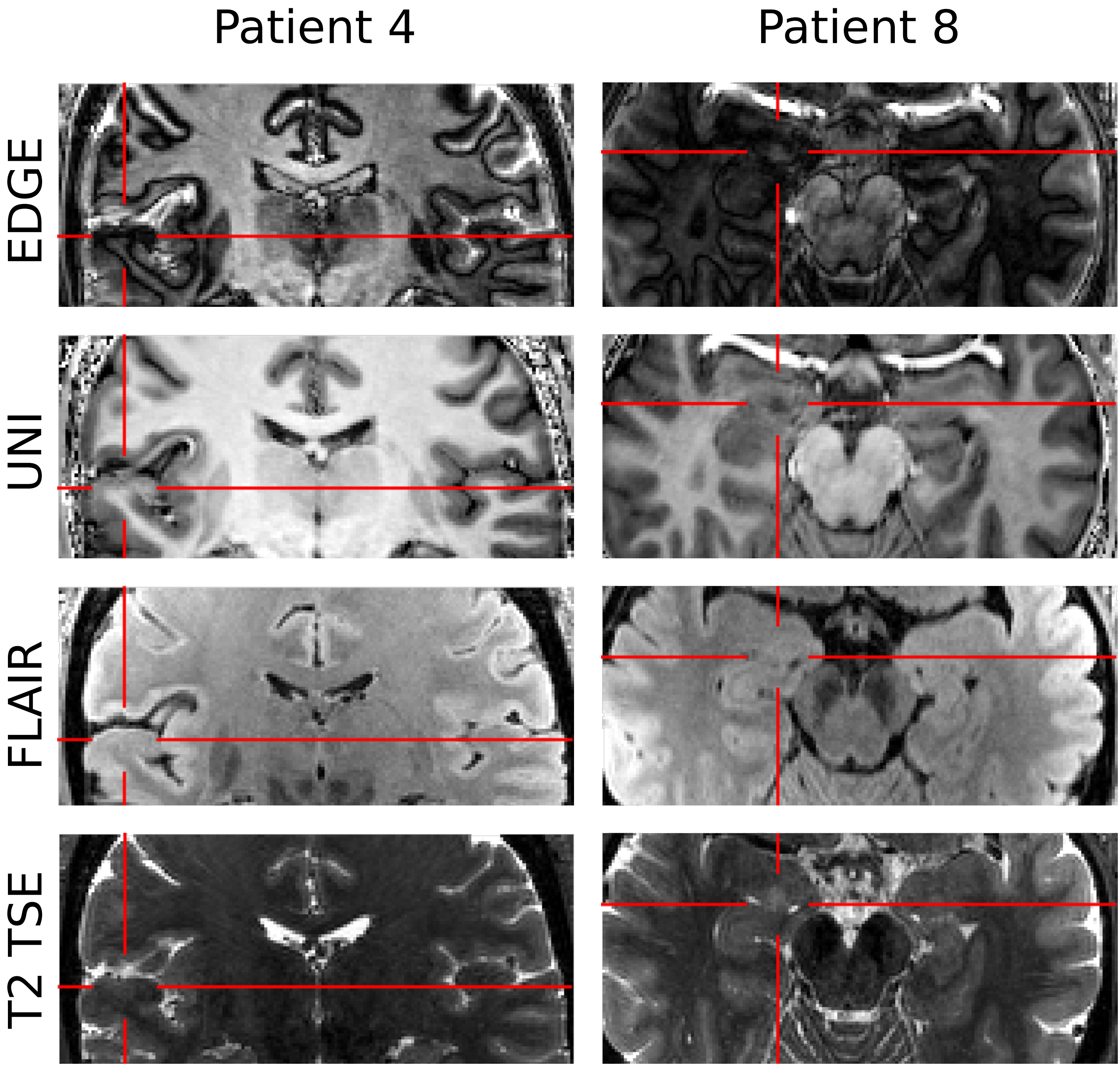

SI 9. High resolution images from Figure 3 part 1.

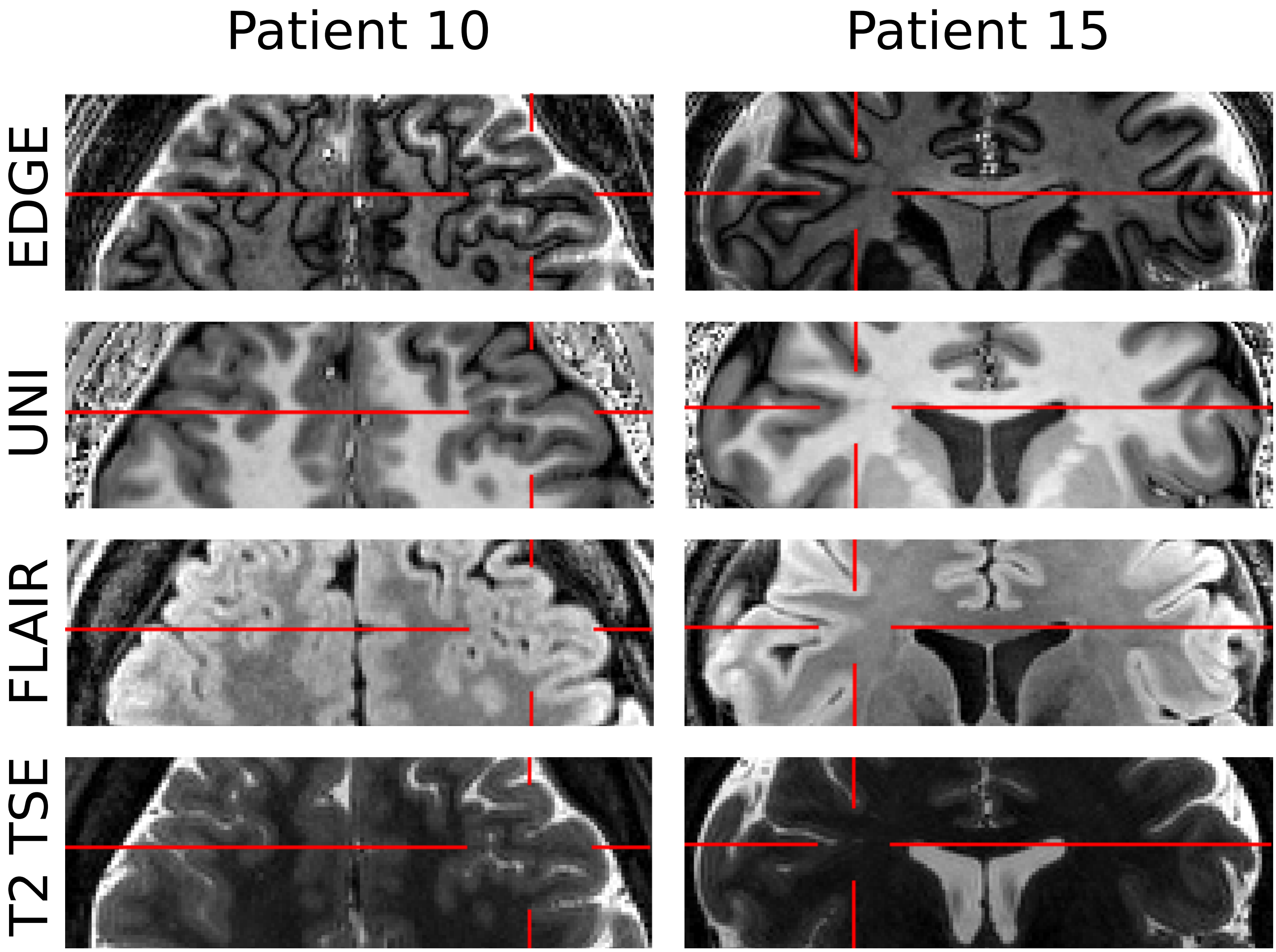

SI 10. High resolution images from Figure 3 part 2.

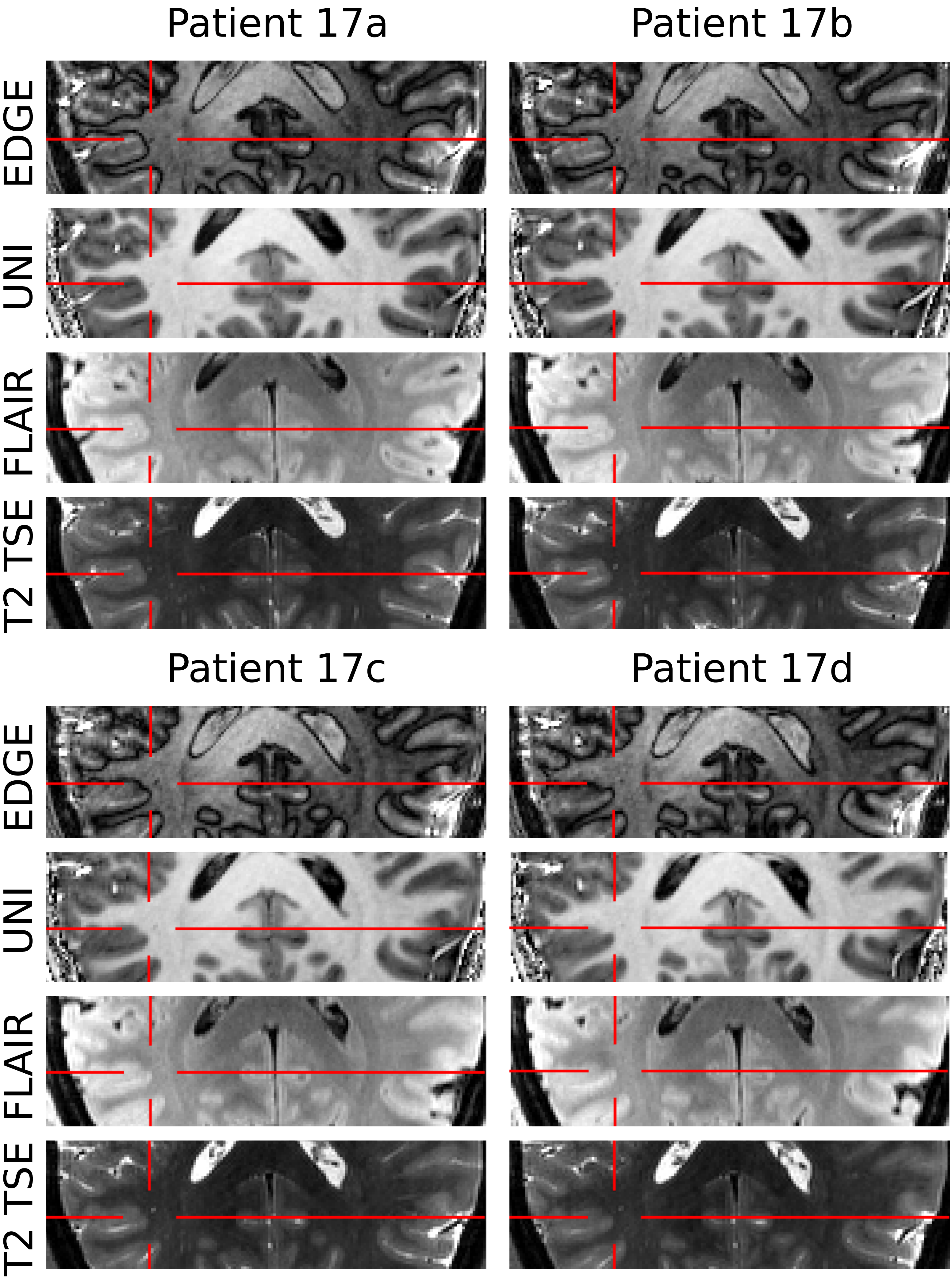

SI 11. High resolution images from Figure 3 part 3.

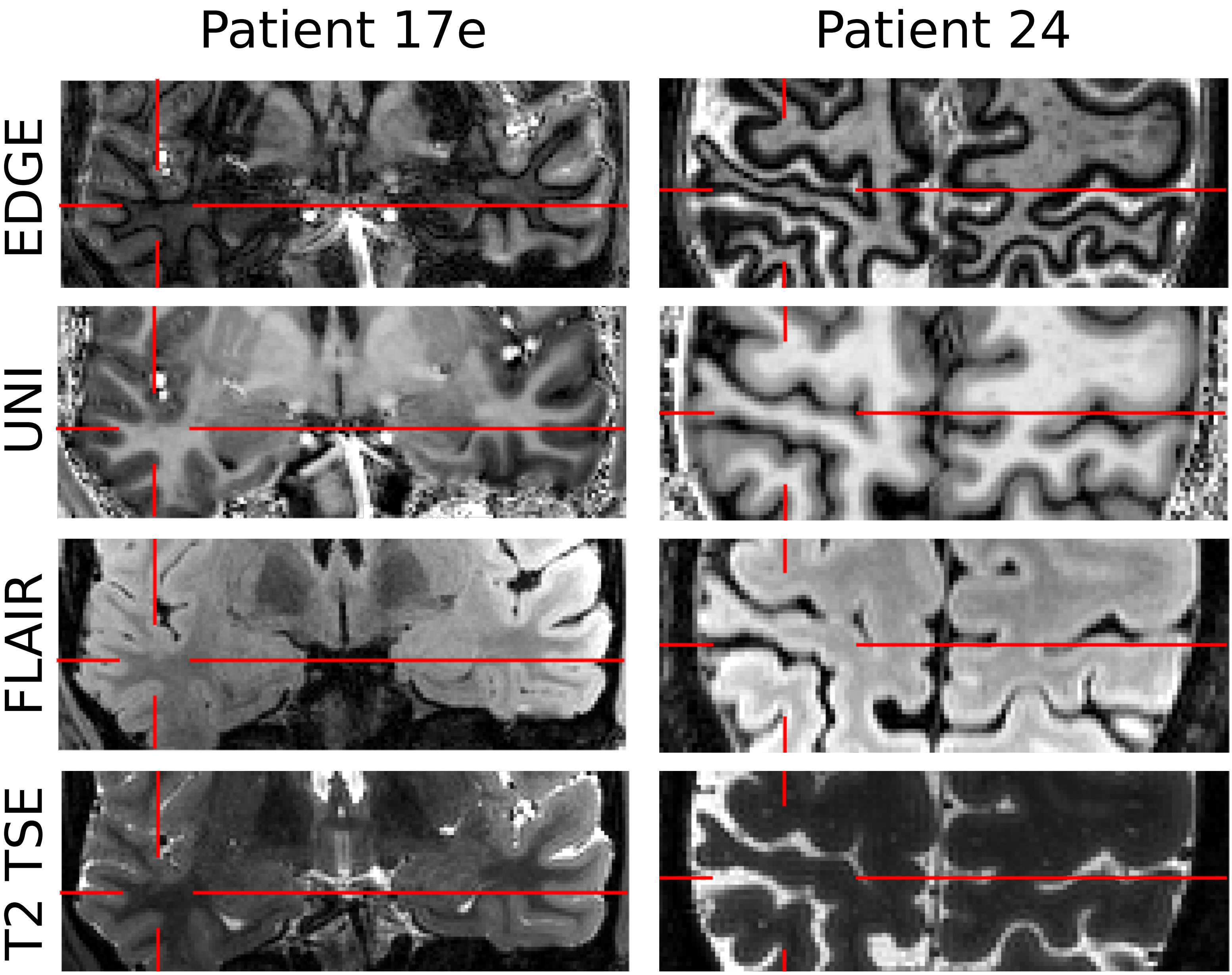

SI 12. High resolution images from Figure 3 part 4.
