# Supplementary material for: Parallel transmit 7T MRI for adult epilepsy pre‐surgical evaluation
Source: Epilepsia. 2025 Mar 20;66(7):2315–27. doi: 10.1111/epi.18353 (PMC12291008; doi:10.1111/epi.18353)
Supplement: Supplementary file 3 — Table S1. [file EPI-66-2315-s003.docx]

Table S1. Image quality blind-assessed by two radiologists (JPJ, DJS), a neurologist (TEC) and a neurosurgeon (RM), using a standardised 1-5 Likert scale^2^: excellent diagnostic quality (5), good diagnostic quality (4), fair diagnostic quality (3), poor diagnostic quality (2), and non-diagnostic quality (1).

| Mean quality rating | pTx FLAIR | CP FLAIR | pTx EDGE | CP EDGE | pTx UNI (T1) | CP UNI (T1) |
| --- | --- | --- | --- | --- | --- | --- |
| Neuroradiologist 1 | 3.75 | 2.33 | 4.53 | 4.53 | 4.33 | 4.33 |
| Neuroradiologist 2 | 3.33 | 2.42 | 3.73 | 3.67 | 4.13 | 4.67 |
| Neurologist | 3.92 | 2.92 | 3.80 | 3.73 | 4.27 | 4.60 |
| Neurosurgeon | 4.08 | 4.50 | 3.80 | 4.00 | 4.00 | 4.07 |

Figure S1: An example comparison set for qualitative pTx vs CP evaluation.

Figure S2: Comparison sequences in the same healthy control subject acquired in a single session with the TI and bandwidth parameters used for our study (‘Optimised’) and with these parameters ‘Flipped’ such that CP sequences were acquired with pTx parameters and vice-versa. This clearly impaired image quality in all cases. Flipped CP UNI images showed poor grey-white-CSF differentiation in the centre of the brain. Flipped CP EDGE images failed to crisply define the grey-white border. Flipped CP FLAIR images showed poor grey-white differentiation and increased noise, particularly in temporal lobes (best appreciated on axial images).
