# Supplementary figures and images for: Parallel transmit 7T MRI for adult epilepsy pre‐surgical evaluation
Source: Epilepsia. 2025 Mar 20;66(7):2315–27. doi: 10.1111/epi.18353 (PMC12291008; doi:10.1111/epi.18353)

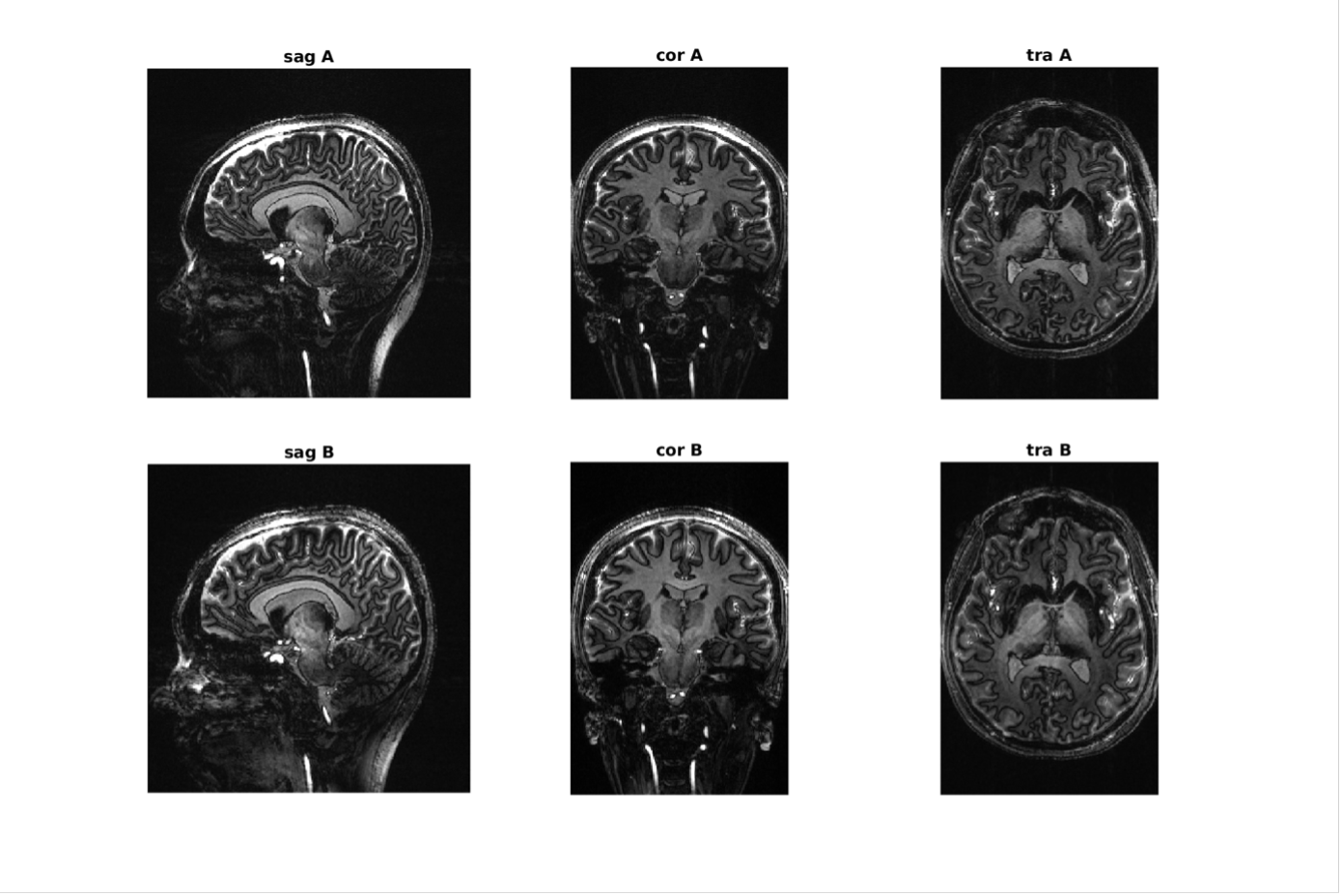

Supplement: Supplementary file 4 — Figure S1. [file EPI-66-2315-s001.tiff]

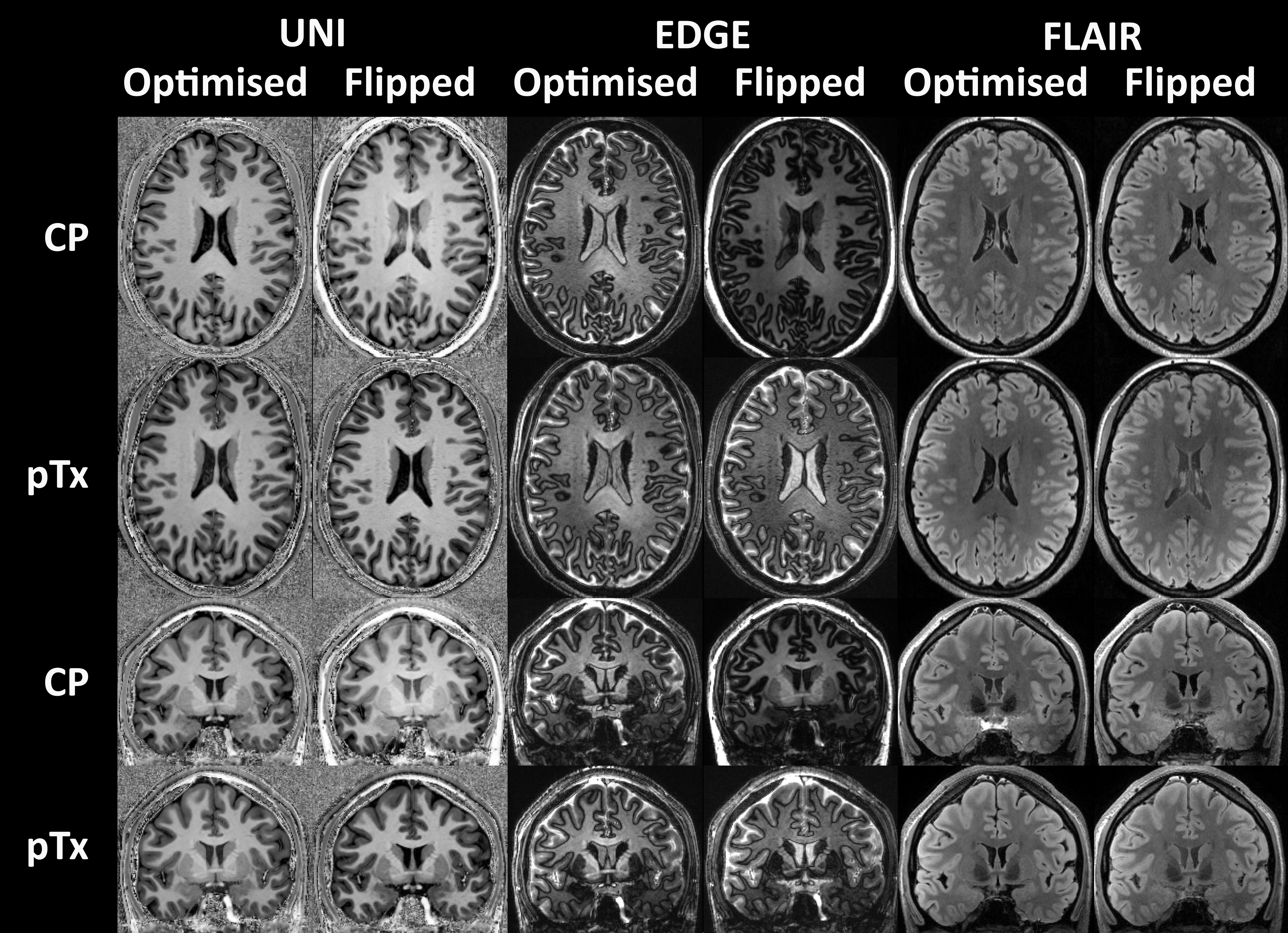

Supplement: Supplementary file 5 — Figure S2. [file EPI-66-2315-s005.png]
